# Neurostructural and Neurophysiological Correlates of Multiple Sclerosis Physical Fatigue: Systematic Review and Meta-Analysis of Cross-Sectional Studies

**DOI:** 10.1007/s11065-021-09508-1

**Published:** 2021-05-07

**Authors:** Paula M. Ellison, Stuart Goodall, Niamh Kennedy, Helen Dawes, Allan Clark, Valerie Pomeroy, Martin Duddy, Mark R. Baker, John M. Saxton

**Affiliations:** 1 Department of Sport, Exercise & Rehabilitation, University of Northumbria, Newcastle upon Tyne, UK; 2 School of Psychology, Ulster University, Coleraine, Northern Ireland, UK; 3 Department of Sport and Health Sciences, Oxford Brookes University, Oxford, UK; 4 Norwich Medical School, University of East Anglia, Norwich, UK; 5 School of Health Sciences and NIHR Brain Injury MedTech Cooperative, University of East Anglia, Norwich, UK; 6 Neurology Department, Royal Victoria Infirmary, Newcastle upon Tyne, UK; 7 Department of Clinical Neurophysiology, Royal Victoria Infirmary, Newcastle upon Tyne, UK; 8 Institute of Neuroscience, Newcastle University, Newcastle upon Tyne, UK; 9 Department of Sport, Health & Exercise Science, University of Hull, Hull, UK

**Keywords:** Multiple sclerosis, Neuroimaging, Neurostructural, Fatigue, Neurophysiology

## Abstract

Fatigue is one of the most debilitating symptoms for people with multiple sclerosis (PwMS). By consolidating a diverse and conflicting evidence-base, this systematic review and meta-analysis aimed to gain new insights into the neurobiology of MS fatigue. MEDLINE, ProQuest, CINAHL, Web of Science databases and grey literature were searched using Medical Subject Headings. Eligible studies compared neuroimaging and neurophysiological data between people experiencing high (MS-HF) versus low (MS-LF) levels of perceived MS fatigue, as defined by validated fatigue questionnaire cut-points. Data were available from 66 studies, with 46 used for meta-analyses. Neuroimaging studies revealed lower volumetric measures in MS-HF versus MS-LF for whole brain (­22.74 ml; 95% CI: -37.72 to -7.76 ml; *p* = 0.003), grey matter (­18.81 ml; 95% CI: ­29.60 to ­8.03 ml; *p* < 0.001), putamen (­0.40 ml; 95% CI: ­0.69 to ­0.10 ml; *p* = 0.008) and acumbens (­0.09 ml; 95% CI: ­0.15 to ­0.03 ml; *p* = 0.003) and a higher volume of T1-weighted hypointense lesions (1.10 ml; 95% CI: 0.47 to 1.73 ml; *p* < 0.001). Neurophysiological data showed reduced lower-limb maximum voluntary force production (­19.23 N; 95% CI: ­35.93 to ­2.53 N; *p* = 0.02) and an attenuation of upper-limb (­5.77%; 95% CI:­8.61 to ­2.93%; *p* < 0.0001) and lower-limb (­2.16%; 95% CI:­4.24 to ­0.07%; *p* = 0.04) skeletal muscle voluntary activation, accompanied by more pronounced upper-limb fatigability (­5.61%; 95% CI: -9.57 to -1.65%; *p* = 0.006) in MS-HF versus MS-LF. Results suggest that MS fatigue is characterised by greater cortico-subcortical grey matter atrophy and neural lesions, accompanied by neurophysiological decrements, which include reduced strength and voluntary activation.

*Prospero registration *Prospero registration number: CRD42016017934

**Supplementary Information:**

The online version contains supplementary material available at 10.1007/s11065-021-09508-1.

## Introduction

Studies show that ≥ 75% of people with multiple sclerosis (PwMS) experience fatigue symptoms persistently or sporadically (Lerdal et al., 2007) and over half the MS population describe it as their most severe symptom (Fisk et al., 1994a). In proposing a unified taxonomy for fatigue in neurologic illness, (Kluger et al., 2013) highlighted the importance of differentiating between *perceived fatigue* and *fatigability* and the application of neuroimaging, neurophysiology and neuropathologic measures to improve our understanding of these two constructs was identified as a research priority. *Perceived fatigue* includes subjective feelings of weariness and an increased subjective perception of effort for everyday tasks (irrespective of recent physical exertion) and is commonly rated in PwMS with the Fatigue Severity Scale (FSS) and Modified Fatigue Impact Scale (MFIS) (Fisk et al., 1994b; Krupp et al., 1989). The FSS is a 9-item scale which focuses on the severity, frequency and impact of fatigue on daily life during the past seven days, whereas the MFIS is a 21-item scale yielding data on the level of cognitive, physical, psychosocial and total fatigue experienced during the past 4 weeks. Validated cut-points of > 4 and ≥ 38 for the FSS (Krupp et al., 1995) and MFIS (Flachenecker et al., 2002), respectively, have been used to classify people experiencing higher (MS-HF) versus lower (MS-LF) levels of perceived MS fatigue. However, other threshold scores have been used as the criterion for higher levels of perceived fatigue in some studies (Dezza et al., 2015; Colombo et al., 2000; Niepel et al., 2006; Tomasevic et al., 2013). In contrast, *fatigability*, sometimes referred to as motor fatigability or performance fatigability, which can include cognitive performance deficits (Kluger et al., 2013; Severijns et al., 2017; Zijdewind et al., 2016), is defined as the rate of change in a performance criterion. Examples include a reduction in maximum voluntary contraction (MVC) force in the motor domain and an objective change in cognitive performance in the cognitive domain. Fatigability during motor and cognitive tasks was divided into central and peripheral components by Kluger et al. (2013). Central fatigue was discussed in terms of central nervous system causes of fatigability, whereas peripheral fatigue relates to fatigability resulting from changes at or distal to the neuromuscular junction (Kluger et al., 2013).

Neuroimaging studies that have investigated associations between perceived fatigue severity and morphometric measures of global brain atrophy or regional atrophy within grey or white matter structures have yielded conflicting results (Gomez et al., 2013; Pellicano et al., 2010; Rocca et al., 2014; Tedeschi et al., 2007). Nevertheless, evidence of impaired functional connectivity between cortical and sub-cortical regions, implicates basal ganglia structures, the thalamus, and specific areas within the frontal, parietal, and temporal lobes in *perceived* MS fatigue (Filippi et al., 2002; Jaeger et al., 2018; Roelcke et al., 1997; Tartaglia et al., 2004; Wilting et al., 2016). Although neuromuscular studies have yielded inconsistent data for voluntary activation (VA) of the upper- and lower-limb skeletal muscles in PwMS versus healthy controls (Andreasen et al., 2009; Ng et al., 2000; Steens et al., 2012a), neurophysiological impairments could underpin the reductions in MVC force (Liepert et al., 2005; Ng et al., 2004; Wolkorte et al., 2016) and motor function (Ng et al., 2004) and the more pronounced levels of fatigability (Liepert et al., 2005; Sheean et al., 1997; Wolkorte et al., 2016) that have been reported in PwMS. Such neurophysiological changes are likely to have a direct bearing on perceived effort for everyday tasks and perceptions of fatigue amongst PwMS.

The aim of this systematic review and meta-analysis was to investigate neurostructural and maladaptive neurophysiological connectivity differences between MS-HF and MS-LF, including motor fatigability. As cognitive functioning is acknowledged as being a complex phenomenon with multifactorial hierarchical domains and numerous approaches to measurement, cognitive performance fatigability was considered to be outside the scope of this research. Current knowledge on the underlying neurobiological substrate of MS fatigue, as assessed by neuroimaging and neurophysiological measures, is impeded by inconsistent findings, insufficiently powered cross-sectional studies and comparisons between healthy controls and PwMS, without partitioning the latter by fatigue status. This makes it difficult to draw definitive conclusions about neurobiological differences between MS-HF and MS-LF and there is a need to consolidate an extensive and somewhat conflicting evidence-base. This systematic review and accompanying meta-analyses addressed these limitations by synthesizing all current evidence, including peer-reviewed (published) neuroimaging and neurophysiological data acquired from senior authors which were not originally presented by fatigue status of PwMS in the published report. By meta-analysing previously reported dichotomised data for MS-HF versus MS-LF, the main aim was to gain an improved insight into structural and neurophysiological correlates of MS fatigue.

## Methods

### Search Strategy

This systematic review and meta-analysis was conducted in accordance with the preferred reporting items for systematic reviews and meta-analyses (PRISMA) statement (Liberati et al., 2009) and the protocol was pre-registered with the PROSPERO International Prospective Register of Systematic Reviews (https://www.crd.york.ac.uk/prospero/display_record.php?RecordID=17934). A systematic literature search of PubMed/MEDLINE, ProQuest, CINAHL and Web of Science from inception until 31 December 2019 was undertaken, blinded to title and authorship, by two reviewers (PE & SG). The search strategy was conducted using Medical Subject Headings (MeSH) and search terms included those related to MS, fatigue, neurophysiology, neuroimaging, MVC, motor nerve stimulation (Supplementary Table 1). We also searched the grey literature (theses, conference abstracts/posters), along with the reference lists of retrieved systematic reviews and included studies to identify other pertinent articles.

### Study Selection

Eligible articles reported data from cross-sectional studies using a validated fatigue scale and defined cut-points for differentiating MS-HF from MS-LF. Adults > 18 years with definite multiple sclerosis (Poser or McDonald criteria) and all types of MS (relapsing–remitting [RR]; secondary progressive [SP]; primary progressive [PP]) were eligible for inclusion. The included studies must have reported neuroimaging measures or neurophysiological variables for MS-HF and MS-LF. However, 14 authors of 16 peer-reviewed published studies provided original data (neurofunctional or neurophysiological) partitioned by *perceived fatigue* status of PwMS (MS-HF versus MS-LF), where it was available but not reported as such in the published article, and these authors have been acknowledged. In the case of the same cohort data being reported in > 1 article, only the most recent publication was included. Non-English animal studies, case studies, review articles, randomised controlled trials and other controlled trials, pharmacological trials and studies that only reported other physical/psychological outcomes (e.g. gait analysis variables, mental health status, disability, cognitive impairment and spiro-ergometric) were excluded.

### Methodological Quality Assessment

The methodological quality of included studies was evaluated using the Cross-Sectional/Prevalence Study Quality Scale, recommended by the Agency for Healthcare Research and Quality (AHRQ: http://www.ncbi.nlm.nih.gov/books/NBK35156/) (Zeng et al., 2015). The AHRQ scale is an 11-item tool that is used to evaluate study quality, with an item scoring “1” if it was answered “Yes” and “0” if it was answered “No”, “Unclear” or “Not Applicable”. Scores of 0–3 indicate “low quality”, 4–7 “moderate quality” and 8–11 “high quality” (Supplementary Table 2). Two reviewers (PE and SG) assessed each included study independently, with disagreements being resolved by consensus and the opinion of a third reviewer (JS).

### Data Extraction and Analysis

Data were extracted independently by three reviewers (PE, SG and JS) as follows: (1) Study design; (2) Characteristics of the participants (number, subtype of MS, disease duration, age, gender and Expanded Disability Status Scale [EDSS] score, fatigue score); (3) Primary outcomes; (4) Secondary outcomes. Means and standard deviations for each variable were extracted for meta-analyses using RevMan 5.0 (http://www.cc-ims.net/RevMan/download.htm). Due to variation in clinical or demographic characteristics and fatigue assessments across studies, a random-effects model was applied throughout, and meta-analyses were not guided by the quality assessment data for individual included studies. A *p* value < 0.05 indicated statistical significance for an overall effect and the magnitude of heterogeneity across studies was tested using the *I*^2^ statistic: *I*^2^ values < 25%, 25–50%, or > 50% indicate low, moderate and high heterogeneity, respectively (Higgins et al., 2003). Funnel plots were not constructed, owing to the number of meta-analyses which included < 10 studies (Sutton et al., 2000). Sub-group analyses were planned based on brain region and limb targeted. Data were not included in meta-analyses if means, standard deviations and number of participants allocated to each group were not reported or available.

## Results

**Fig. 1:**
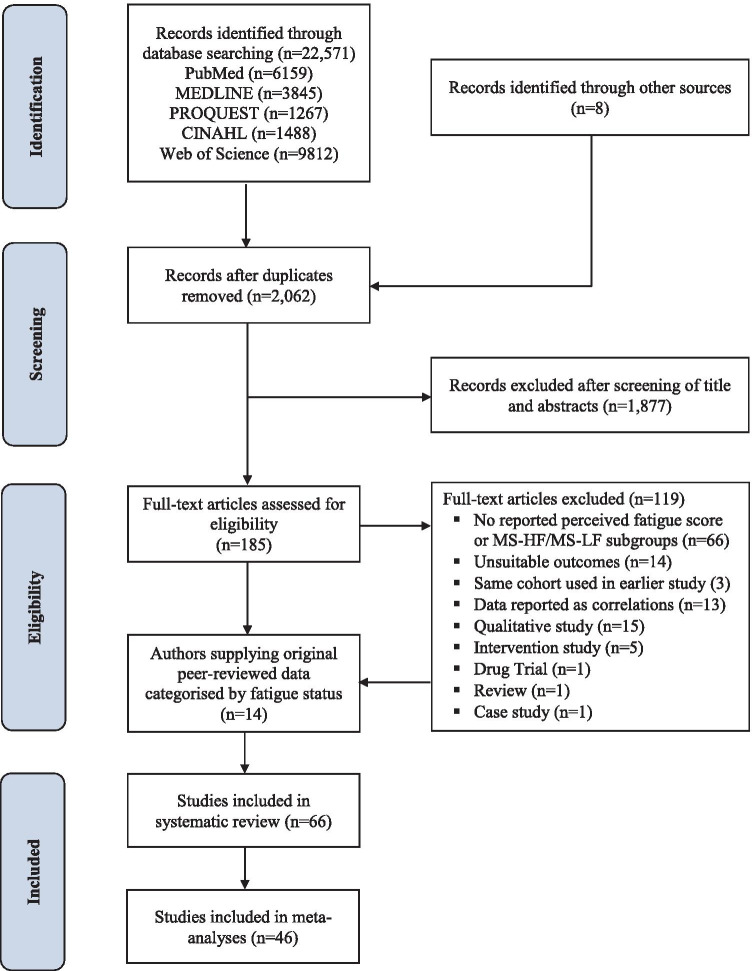
PRISMA flow chart for literature search and study selection

Figure 1 shows that the search yielded 66 studies, with data from 46 studies included in meta-analyses (42 neuroimaging studies, 19 neurophysiological studies and five combined neuroimaging and neurophysiological studies). Supplementary Tables 3a and 3b present details of each included study. A total of 1761 MS-HF and 1391 MS-LF participants were compared in the cross-sectional studies, with the majority (2345) having a definite RRMS diagnosis, 150 being classified as PPMS or SPMS and 575 participants of unspecified disease type. In 48 studies, healthy controls were included as an additional comparison. Studies which provided details of the gender balance for MS-HF and MS-LF (N = 43) showed there were approximately twice as many women than men in each subgroup (729:357 and 657:387, respectively), reflecting the higher prevalence of MS amongst women (Harbo et al., 2013). The MS-HF and MS-LF groups were well-balanced for age, disease duration and EDSS score. The mean age was 40 years for MS-HF versus 38 years for MS-LF (reported in 56 studies). MS-HF had a mean disease duration (years) and EDSS score of 8.7 years (reported in 41 studies) and 2.6 (reported in 52 studies) respectively, versus 8.1 years and 2.0, respectively for MS-LF. EDSS scores indicated a mild to moderate level of disability with no impairment to walking for the majority of MS-HF and MS-LF participants (EDSS ≤ 3.5 in 85% of studies).

**Table 1:** Summary of the results of meta-analyses for neuroimaging and neurophysiological studies (MS-HF versus MS-LF)

Variable	Number of studies	Number of Participants		Mean difference (95% CI)	*P*	Heterogeneity
		MS-HF	MS-LF			
*Neuroimaging variables*						
**Mean normalised brain volume (ml)**	**11**	**336**	**375**	**-22.74 (-37.72, -7.76)**	**0.003**	**χ**^**2**^** = 7.24; p = 0.70; I**^**2**^** = 0%**
Brain parenchymal fraction (%)	6	129	159	0.17 (-0.54, 0.88)	0.64	χ^2^ = 3.03; p = 0.70; I^2^ = 0%
**Grey matter volume (ml)**	**9**	**306**	**318**	**-18.81 (-29.60, -8.03)**	**0.0006**	**χ**^**2**^** = 5.71; p = 0.68; I**^**2**^** = 0%**
White matter volume (ml)	9	306	318	-6.41 (-13.98, 1.15)	0.10	χ^2^ = 2.94; p = 0.94; I^2^ = 0%
Thalamus volume (ml)	8	234	286	-0.56 (-1.44, 0.31)	0.21	χ^2^ = 88.55; p < 0.00001; I^2^ = 92%
**Putamen volume (ml)**	**4**	**163**	**178**	**-0.40 (-0.69, -0.10)**	**0.008**	**χ**^**2**^** = 4.89; p = 0.18; I**^**2**^** = 39%**
Caudate volume (ml)	4	163	178	-0.45 (-0.95, 0.04)	0.07	χ^2^ = 27.43; p < 0.00001; I^2^ = 89%
**Accumbens volume (ml)**	**2**	**53**	**59**	**-0.09 (-0.15, -0.03)**	**0.003**	**χ**^**2**^** = 0.36; p = 0.55; I**^**2**^** = 0%**
Amygdala volume (ml)	2	53	59	-0.00 (-0.15, 0.14)	0.95	χ^2^ = 1.27; p = 0.27; I^2^ = 19%
Pallidus volume (ml)	2	46	46	-0.23 (-0.50, 0.04)	0.09	χ^2^ = 2.90; p = 0.09; I^2^ = 66%
**T1-weighted Lesion volume (ml)**	**9**	**483**	**334**	**1.10 (0.47, 1.73)**	**0.0007**	**χ**^**2**^** = 8.90; p < 0.35; I**^**2**^** = 10%**
T2-weighted lesion volume (ml)	21	730	596	1.19 (-0.43, 2.80)	0.15	χ^2^ = 42.25; p < 0.003; I^2^ = 53%
Fractional anisotrophy	3	60	60	-0.01 (-0.02, 0.01)	0.29	χ^2^ = 8.99; p = 0.01; I^2^ = 78%
Mean diffusivity (× 10^−3^ mm^2^/s)	3	60	60	0.01 (-0.03, 0.05)	0.72	χ^2^ = 9.04; p = 0.01; I^2^ = 78%
NAA/Cr ratio	3	67	56	-0.12 (-0.27, 0.03)	0.11	χ^2^ = 7.63; p = 0.02; I^2^ = 74%
Cho/Cr ratio	2	51	39	-0.02 (-0.09, 0.04)	0.48	χ^2^ = 0.34; p = 0.56; I^2^ = 0%
*Neurophysiological variables*						
**Upper-limb MVC (N)**	**6**	**130**	**69**	**-3.55 (-7.11, 0.01)**	**0.05**	**χ**^**2**^** = 3.23; p = 0.66; I**^**2**^** = 0%**
**Lower-limb MVC (N)**	**4**	**72**	**55**	**-19.23 (-35.93, -2.53)**	**0.02**	**χ**^**2**^** = 2.43; p = 0.49; I**^**2**^** = 0%**
**Upper-Limb voluntary activation (%)**	**3**	**33**	**29**	**-5.77 (-8.61, -2.93)**	** < 0.0001**	**χ**^**2**^** = 0.45, p = 0.80; I**^**2**^** = 0%**
**Lower-limb voluntary activation (%)**	**3**	**36**	**29**	**-2.16 (-4.24, -0.07)**	**0.04**	**χ**^**2**^** = 0.11; p = 0.94; I**^**2**^** = 0%**
Motor evoked potential threshold (%)	3	61	34	-0.05 (-5.46, 5.36)	0.99	χ^2^ = 3.09; p = 0.21; I^2^ = 35%
Motor evoked potential amplitude (mV)	2	40	17	-0.09 (-0.42, 0.23)	0.57	χ^2^ = 1.18; p = 0.28; I^2^ = 15%
Motor evoked potential latency (ms)	2	40	17	1.70 (-2.09, 5.50)	0.38	χ^2^ = 5.21; p = 0.02; I^2^ = 81%
Central motor conduction time (ms)	2	32	19	-0.74 (-2.75, 1.27)	0.47	χ^2^ = 0.05; p = 0.82; I^2^ = 0%
Short interval intracortical inhibition (%)	3	45	42	-1.06 (-30.08, 27.96)	0.94	χ^2^ = 10.64; p = 0.005; I^2^ = 81%
Intracortical facilitation (%)	3	45	42	1.74 (-18.36, 21.84)	0.87	χ^2^ = 0.72; p = 0.70; I^2^ = 0%
**Upper-limb post-fatigue task MVC (%)**	**5**	**139**	**87**	**-5.61 (-9.57, -1.65)**	**0.006**	**χ**^**2**^** = 5.04; p = 0.28; I**^**2**^** = 21%**

Data are presented as absolute mean differences with 95% confidence intervals

*MVC* maximum voluntary contraction force

*P<*0.05

**Table 2:** Summary of the results of meta-analyses for neuroimaging and neurophysiological studies (MS-HF versus HC)

Variable	Number of studies	Number of Participants		Mean difference (95% CI)	*P*	Heterogeneity
		MS-HF	HC			
*Neuroimaging variables*						
**Mean normalised brain volume (ml)**	**9**	305	356	**-74.01 (-88.86, -59.16)**	** < 0.00001**	**χ**^**2**^** = 7.80; p = 0.45; I**^**2**^** = 0%**
**Brain parenchymal fraction (%)**	**5**	**118**	**90**	**-2.06 (-3.12, -0.99)**	**0.0002**	**χ**^**2**^** = 5.65; p = 0.23; I**^**2**^** = 29%**
**Grey matter volume (ml)**	**8**	290	343	**-58.96 (-79.21, -38.72)**	** < 0.00001**	**χ**^**2**^** = 27.73; p = 0.0002; I**^**2**^** = 75%**
**White matter volume (ml)**	**8**	290	343	**-33.22 (-44.28, -22.15)**	** < 0.00001**	**χ**^**2**^** = 15.68; p = 0.03; I**^**2**^** = 55%**
**Thalamus volume (ml)**	**6**	208	235	**-1.67 (-2.25, -1.09)**	** < 0.00001**	**χ**^**2**^** = 28.00; p < 0.0001; I**^**2**^** = 82%**
**Putamen volume (ml)**	**4**	163	148	**-1.07 (-1.50, -0.63)**	** < 0.00001**	**χ**^**2**^** = 10.82; p = 0.01; I**^**2**^** = 72%**
**Caudate volume (ml)**	**4**	163	145	**-0.84 (-1.15, -0.53)**	** < 0.00001**	**χ**^**2**^** = 8.95; p = 0.03; I**^**2**^** = 66%**
**Accumbens volume (ml)**	**2**	**53**	**59**	**-0.17 (-0.34, -0.01)**	**0.04**	**χ**^**2**^** = 4.19; p < 0.04; I**^**2**^** = 76%**
Amygdala volume (ml)	2	53	59	-0.10 (-0.56, 0.36)	0.67	χ^2^ = 7.08; p < 0.008; I^2^ = 86%
**T1-weighted lesion volume (ml)**	**2**	49	48	**4.66 (2.42, 6.90)**	** < 0.0001**	**χ**^**2**^** = 3.42; p = 0.06; I**^**2**^** = 71%**
Fractional anisotrophy	2	45	65	-0.02 (-0.04, 0.01)	0.31	χ^2^ = 25.86; p < 0.00001; I^2^ = 96%
**Mean diffusivity (× 10**^**−3**^** mm**^**2**^**/s)**	**2**	**45**	**65**	**0.02 (0.01, 0.03)**	**0.0009**	**χ**^**2**^** = 0.00; p = 1.00; I**^**2**^** = 0%**
**NAA/Cr ratio**	**2**	33	27	**-0.10 (-0.18, -0.01)**	**0.03**	**χ**^**2**^** = 0.01; p = 0.91; I**^**2**^** = 0%**
*Neurophysiological variables*						
**Upper-limb MVC (N)**	**5**	69	82	**-8.73 (-16.71, -0.75)**	**0.03**	**χ**^**2**^** = 9.01; p = 0.06; I**^**2**^** = 56%**
**Lower-limb MVC (N)**	**2**	17	29	**-63.94 (-128.18, 0.31)**	**0.05**	**χ**^**2**^** = 1.91; p = 0.17; I**^**2**^** = 48%**
**Motor evoked potential threshold (%)**	**2**	40	19	**8.46 (2.73, 14.18)**	**0.004**	**χ**^**2**^** = 1.75; p = 0.19; I**^**2**^** = 43%**
Motor evoked potential amplitude (mV)	2	40	19	-0.74 (-2.13, 0.65)	0.30	χ^2^ = 7.28; p = 0.007; I^2^ = 86%
Motor evoked potential latency (ms)	2	40	19	2.81 (-2.09, 7.71)	0.26	χ^2^ = 14.24; p = 0.0002; I^2^ = 93%
Short interval intracortical inhibition (%)	2	24	18	11.93 (-10.99, 34.86)	0.31	χ^2^ = 2.09; p = 0.15; I^2^ = 52%
Intracortical facilitation (%)	2	24	18	1.67 (-22.96, 26.30)	0.89	χ^2^ = 1.17; p = 0.28; I^2^ = 15%
**Upper-limb post-fatigue task MVC (%)**	**4**	78	109	**-7.43 (-11.95, -2.90)**	**0.001**	**χ**^**2**^** = 4.28; p = 0.23; I**^**2**^** = 30%**

Data are presented as absolute mean differences with 95% confidence intervals

*MVC* maximum voluntary contraction force

Significance: *P<*0.05

**Table 3:** Summary of the results of meta-analyses for neuroimaging and neurophysiological studies (MS-LF versus HC)

Variable	Number of studies	Number of Participants		Mean difference (95% CI)	*P*	Heterogeneity
		MS-LF	HC			
*Neuroimaging variables*						
**Mean normalised brain volume (ml)**	**9**	333	356	**-51.59 (-71.80, -31.38)**	** < 0.00001**	**χ**^**2**^** = 15.57; p = 0.05; I**^**2**^** = 49%**
**Brain parenchymal fraction (%)**	**5**	150	90	**-1.95 (-3.46, -0.44)**	**0.01**	**χ**^**2**^** = 15.27; p = 0.004; I**^**2**^** = 74%**
**Grey matter volume (ml)**	**8**	301	343	**-41.39 (-62.63, -20.16)**	**0.0001**	**χ**^**2**^** = 32.39; p < 0.0001; I**^**2**^** = 78%**
**White matter volume (ml)**	**8**	301	343	**-25.51 (-37.27, -13.76)**	**0.0001**	**χ**^**2**^** = 16.43; p = 0.02; I**^**2**^** = 57%**
**Thalamus volume (ml)**	**6**	263	235	**-1.10 (-2.13, -0.07)**	**0.04**	**χ**^**2**^** = 103.20; p < 0.00001; I**^**2**^** = 95%**
**Putamen volume (ml)**	**4**	178	148	**-0.65 (-0.93, -0.38)**	** < 0.00001**	**χ**^**2**^** = 5.01; p = 0.17; I**^**2**^** = 40%**
**Caudate volume (ml)**	**4**	178	148	**-0.36 (-0.66, -0.06)**	**0.02**	**χ**^**2**^** = 8.48; p = 0.04; I**^**2**^** = 65%**
Accumbens volume (ml)	2	53	59	-0.10 (-0.31, 0.11)	0.36	χ^2^ = 7.18; p = 0.007; I^2^ = 86%
Amygdala volume (ml)	2	53	59	-0.03 (-0.29, 0.24)	0.85	χ^2^ = 3.06; p = 0.08; I^2^ = 67%
**T1-weighted lesion volume (ml)**	**2**	56	48	**5.81 (3.93, 7.69)**	** < 0.00001**	**χ**^**2**^** = 0.18; p = 0.68; I**^**2**^** = 0%**
**Fractional anisotrophy**	**2**	46	65	**-0.02 (-0.04, -0.00)**	**0.04**	**χ**^**2**^** = 11.63; p = 0.0007; I**^**2**^** = 91%**
**Mean diffusivity (× 10**^**−3**^** mm**^**2**^**/s)**	**2**	**46**	**65**	**0.03 (0.00, 0.06)**	**0.03**	**χ**^**2**^** = 3.79; p = 0.05; I**^**2**^** = 74%**
NAA/Cr ratio	2	30	27	-0.05 (-0.12, 0.02)	0.19	χ^2^ = 0.07; p = 0.79; I^2^ = 0%
*Neurophysiological variables*						
**Upper-limb MVC (N)**	**5**	48	82	**-5.33 (-8.79, -1.86)**	**0.003**	**χ**^**2**^** = 2.65; p = 0.62; I**^**2**^** = 0%**
Lower-limb MVC (N)	2	8	29	-74.31 (-166.56, 17.93)	0.11	χ^2^ = 4.21; p = 0.04; I^2^ = 76%
**Motor evoked potential threshold (%)**	**2**	17	19	**5.60 (1.02, 10.18)**	**0.02**	**χ**^**2**^** = 0.04; p = 0.84; I**^**2**^** = 0%**
Motor evoked potential amplitude (mV)	2	17	19	-0.33 (-1.15, 0.48)	0.42	χ^2^ = 2.03; p = 0.15; I^2^ = 51%
Motor evoked potential latency (ms)	2	17	19	0.67 (-0.62, 1.96)	0.31	χ^2^ = 0.51; p = 0.47; I^2^ = 0%
Short interval intracortical inhibition (%)	2	25	18	0.58 (-10.37, 11.53)	0.92	χ^2^ = 1.04; p = 0.31; I^2^ = 4%
Intracortical facilitation (%)	2	25	18	3.90 (-40.99, 48.79)	0.86	χ^2^ = 3.55; p = 0.06; I^2^ = 72%
Upper-limb post-fatigue task MVC (%)	4	49	109	-2.91 (-6.78, 0.96)	0.14	χ^2^ = 1.49; p = 0.68; I^2^ = 0%

Data are presented as absolute mean differences with 95% confidence intervals

*MVC* maximum voluntary contraction force

Significance: *P<*0.05

### Perceived Fatigue Measures

The most frequently used scale to differentiate MS-HF from MS-LF was the FSS (Krupp et al., 1989), which was used in 48 of the included studies, using mean cut-off scores for MS-HF of > 4 or > 5 and total scores ranging from > 25 to > 36. A further 10 studies used the MFIS (Fisk et al., 1994a) with cut-off scores for MS-HF in the range of > 35 to > 38 or ≥ 16 for the MFIS physical scale. Three studies used the cognitive scale of the Fatigue Scale for Motor and Cognitive Functions using cut-points in the range of ≥ 22 to ≥ 28 (Pravata et al., 2016; Sander et al., 2016; Wilting et al., 2016). These studies were included because they reported regional brain volume or functional connectivity data and two of them provided evidence that high levels of cognitive fatigue are accompanied by higher levels of motor fatigue (Pravata et al., 2016; Sander et al., 2016). Two further studies used the MFIS-5 and EMIF-SEP (a validated French version of the Fatigue Impact Scale) and three studies used subjective perceptions to classify MS-HF, e.g. “mostly or daily tired” (Supplementary Tables 3a and 3b).

### Neuroimaging and Neurophysiological Measures

Neuroimaging measures for meta-analyses were obtained using magnetic resonance imaging (MRI), diffusion tensor imaging (DTI) and magnetic resonance spectroscopy (MRS). Measures included total normalised brain volume, grey and white matter volumes, T1-weighted hypointense and T2-weighted lesion volumes, white matter microstructural integrity (DTI indices of fractional anisotropy and mean diffusivity) and neuronal/axonal integrity and function (N-acetylaspartate to creatine [NAA/Cr] ratio and choline to creatine [Cho/cr] ratio by MRS). Neurophysiological measures for meta-analyses were obtained using transcranial magnetic stimulation (TMS), electroencephalography (EEG), neuromuscular electrical stimulation (NMES) and electromyography (EMG) and included motor evoked potential (MEP) amplitude, MEP latency, MEP threshold, central motor conduction time, short-interval intracortical inhibition (SICI), and voluntary activation (central motor drive) using the twitch-interpolation technique during MVC (Merton, 1954). Brain region functional connectivity data determined using functional MRI (fMRI) were not included in meta-analyses but the key findings are reported in Supplementary Table 3a. MVC force data were determined using upper- or lower-limb rigs that fixed the joint in position for isometric muscle actions, with motor fatigability being assessed using a sustained MVC or intermittent %MVC isometric fatiguing protocol and reported as percent of the baseline force.

### Meta-Analyses Overview

**Fig. 2:**
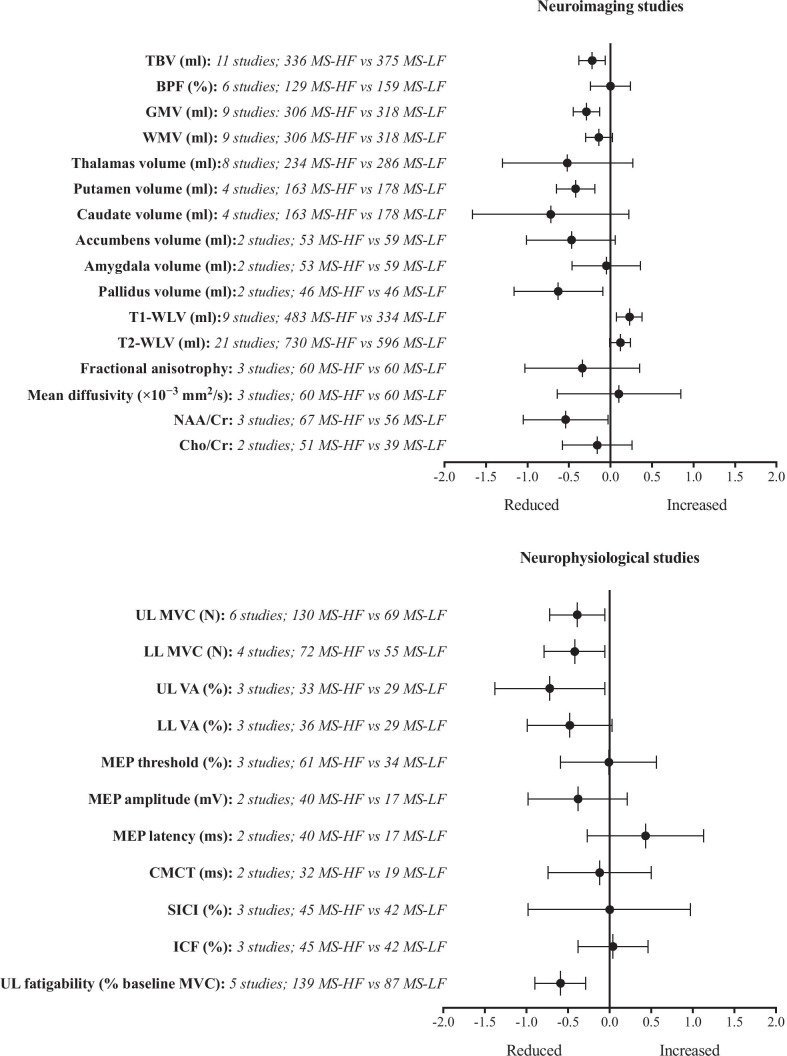
Summary of results of meta-analyses comparing neuroimaging and neurofunctional data for MS-HF versus MS-LF. Data are presented as standardised mean difference and 95% confidence intervals. The upper figure presents summary data for neuroimaging variables and the lower figure presents summary data for neurofunctional variables, with the abscissas representing a decrease or increase for MS-HF in comparison with MS-LF. TBV, total brain volume; BPF, brain parenchymal fraction; GMV, gray matter volume, WMV, white matter volume, T1-WLV, T1-weighted lesion volume, T2-WLV, T2-weighted lesion volume, NAA/Cr, N-acetylaspartate to creatine ratio Cho/Cr, choline to creatine ratio, UL, upper-limb; LL, lower-limb; MEP, motor evoked potential; CMCT, central motor conduction time; SICI, short-interval intracortical inhibition, ICF, intracortical facilitation; MVC, maximum voluntary contraction force

The results of meta-analyses are presented as absolute mean differences with 95% confidence intervals (CI) in Table 1. Table 1 also presents the number of studies and number of participants in the MS-HF and MS-LF groups for each meta-analysis. Detailed forest plots showing comparisons of MS-HF versus MS-LF, MS-HF versus HC and MS-LF versus HC are presented in Supplementary Figs. 1, 2 and 3. A summary of meta-analyses results for all neuroimaging and neurophysiological variables (MS-HF versus MS-LF) are presented as standardised mean difference (SMD) and 95% CI in Fig. 2, with Cohen’s categories (SMD = 0.2–0.5; 0.5–0.8; ≥ 0.8) indicating small, medium and large overall effect sizes, respectively.

## Quality Assessment

**Fig. 3:**
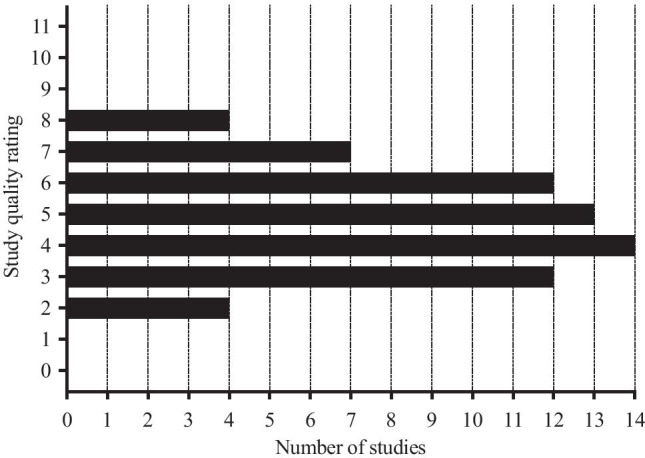
Methodological quality of the included studies evaluated using the Cross-Sectional/Prevalence Study Quality Scale, recommended by the Agency for Healthcare Research and Quality (AHRQ). Scores of 0–3 indicate “low quality”, 4–7 “moderate quality” and 8–11 “high quality”

Most of the included studies (70%) were classified as being of “moderate quality”, 16 (24%) studies were rated as “low quality” and four studies (6%) as “high quality” (Fig. 3; Supplementary Table 2). Key limitations representing risk of bias included inadequate details of the time period used to identify and recruit participants, use of non-blinded evaluators and lack of quality control data for the methods used to compare MS-HF with MS-LF.

## Neuroimaging Meta-Analyses

### Brain Volume

Meta-analysis suggested a reduction in mean normalised brain volume (­22.74 ml; 95% CI: -37.72 to -7.76 ml; *p* = 0.003) in MS-HF versus MS-LF, accompanied by a reduction in the volume of grey matter in MS-HF versus MS-LF (­18.81 ml; 95% CI: ­29.60 to ­8.03 ml; *p* < 0.001). There was no significant difference in white matter volume between MS-HF and MS-LF (-6.41 ml; 95% CI: -13.98 to 1.15 ml; *p* = 0.10). Larger reductions in mean normalised brain volume, grey and white matter volumes (all *p* < 0.001) were apparent for MS-LF and MS-HF versus HC (Tables 1, 2 and 3; Supplementary Figs. 1, 2, and 3).

### Subcortical Grey Matter Structure Volumes

Where data for sub-cortical structures were reported for the left and right sides, data were summed to provide a single volumetric measure for comparison with studies in which a single volumetric measure was reported. Meta-analysis showed a reduction in putamen (­0.40 ml; 95% CI: ­0.69 to ­0.10 ml; *p* = 0.008) and accumbens (­0.09 ml; 95% CI: ­0.15 to ­0.03 ml; *p* = 0.003) volumes for MS-HF versus MS-LF. Larger effect-size reductions in thalamus and caudate volumes did not reach statistical significance because of wider confidence intervals and there were high levels of heterogeneity (*I*^2^ ≥ 89%; Fig. 2). Volumetric reductions were apparent for the thalamus, putamen and caudate (*p* ≤ 0.02) in MS-LF and MS-HF versus HC, and for the accumbens in MS-HF versus HC (*p* = 0.04; Tables 1, 2 and 3; Supplementary Figs. 1, 2, and 3).

### Lesion Volume, White Matter and Axonal Integrity and Function

There was an increased volume of T1-weighted hypointense lesions in MS-HF versus MS-LF (1.10 ml; 95% CI: 0.47 to 1.73 ml; *p* < 0.001) and for MS-LF and MS-HF versus HC (*p* < 0.0001). However, there were no differences between MS-HF and MS-LF for T2-weighted lesion volume (1.19 ml; 95% CI: -0.43 to 2.80 ml; *p* = 0.15), white matter microstructural integrity (DTI indices of fractional anisotropy and mean diffusivity) or axonal integrity/function (NAA/Cr or Cho/Cr by MRS). There was an increase in DTI mean diffusivity for MS-HF (0.02 × 10^−3^ mm^2^/s; 95% CI: 0.01 to 0.03 × 10^−3^ mm^2^/s; *p* < 0.001) and MS-LF (0.03 × 10^−3^ mm^2^/s; 95% CI: 0.00 to 0.06 × 10^−3^ mm^2^/s; *p* = 0.03) versus HC, and a reduction in the NAA/Cr ratio in MS-HF versus HC (-0.10; 95% CI: -0.18 to -0.01; *p* < 0.03), indicating relative impairment of microstructural and axonal integrity/function (Tables 1, 2 and 3; Supplementary Figs. 1, 2, and 3).

## Neurophysiological Meta-Analyses

### Corticospinal Integrity and Intra-Cortical Inhibition

There were no significant differences between MS-HF and MS-LF in MEP amplitude, latency, threshold, central motor conduction time or SICI. However, higher MEP thresholds were apparent for MS-LF and MS-HF versus HC (*p* ≤ 0.02; Tables 1, 2 and 3; Supplementary Figs. 1, 2, and 3).

### Skeletal Muscle Maximum Voluntary Contraction Force and Voluntary Activation

There were reductions in lower-limb (­-19.23 N; 95% CI: -­35.93 to -­2.53 N; *p* = 0.02) and upper-limb MVC force (-­3.55 N; 95% CI: -­7.11 to 0.01 N; *p* = 0.05) in MS-HF versus MS-LF, with the latter of borderline statistical significance. Reductions in upper-limb MVC force were also apparent in MS-LF and MS-HF versus HC (*p* ≤ 0.03). Meta-analysis of studies which used the twitch-interpolation technique during a MVC showed reduced voluntary activation in MS-HF versus MS-LF for upper-limb (­5.77%; 95% CI:­8.61 to ­2.93%; *p* < 0.0001) and lower-limb skeletal muscles (­-2.16%; 95% CI:­-4.24 to -­0.07%; *p* = 0.04). Upper-limb muscles included finger and thumb flexors/extensors and lower-limb muscles included the quadriceps and dorsiflexors (Tables 1, 2 and 3; Supplementary Figs. 1, 2, and 3).

### Motor Fatigability

Meta-analysis of the percent decline in MVC from baseline after an upper-limb (finger or thumb flexor/extensor) skeletal muscle fatigue task (either sustained [N = 3] or intermittent [N = 2] isometric MVC) revealed greater motor fatigability for MS-HF versus MS-LF (­5.61%; 95% CI: -9.57 to -1.65%; *p* = 0.006). A more pronounced level of upper-limb motor fatigability was also observed for MS-HF versus HC (­7.43%; 95% CI: -11.95 to -2.90%; *p* = 0.001; Tables 1, 2 and 3; Supplementary Figs. 1, 2, and 3).

## Discussion

### Overview

Using a dichotomised model (MS-HF versus MS-LF), this systematic review and accompanying meta-analyses aimed to provide an improved insight into structural and neurophysiological correlates of MS fatigue. By robustly consolidating an extensive and somewhat conflicting evidence-base, the results suggest that higher levels of MS fatigue are characterised by greater cortico-subcortical atrophy, and with indications of greater neural damage, as evidenced by an increased volume of T1-weighted hypointense lesions (Napoli & Bakshi, 2005). These neurostructural impairments appear to be accompanied by neurophysiological decrements, manifest as impaired MVC force and reduced skeletal muscle voluntary activation. As most studies were categorised as being of moderate quality, and also considering there were differences in the exact cut-points used to classify fatigue status across different studies, some level of caution is required when interpreting meta-analysis results. Nevertheless, the synthesis of available cross-sectional data from the included studies, together with published peer-reviewed data (acquired from senior authors) that were not originally presented by fatigue status of PwMS, means these results provide the most precise effect-size estimates of neurobiological differences between MS-HF and MS-LF to date.

### Global and Regional Brain Volumes

Although the meta-analyses provided clear evidence of white matter atrophy in MS-HF and MS-LF versus HC, the smaller normalised brain volume in MS-HF versus MS-LF appears to be mainly attributable to a volumetric reduction in grey matter. Cortical regions with reduced volumes for MS-HF versus MS-LF in the included studies were the precentral gyrus, inferior and superior temporal gyrus, superior and inferior frontal gyrus, anterior cingulate gyrus, central sulcus, superior and inferior parietal lobules (Andreasen et al., 2010; Riccitelli et al., 2011; Rocca et al., 2014; Sepulcre et al., 2009). This meta-analysis also consolidated the evidence for sub-cortical grey matter structures, revealing volumetric reductions in the putamen and accumbens for MS-HF versus MS-LF. Evidence suggests that putamen atrophy is present early in the MS disease cycle (Kramer et al., 2015) and many participants recruited to the included studies are likely to have fallen into this category (EDSS ≤ 3.5 in over 80% of studies). Interestingly, larger effect size reductions in caudate and thalamus volumes were also observed in MS-HF versus MS-LF and HC but these only reached statistical significance for comparisons with the HC data. Other sub-cortical and basal ganglia structures reported to have reduced volumes in MS-HF versus MS-LF which were inversely correlated with perceived fatigue were the pallidum and superior cerebellar peduncle (Bernitsas et al., 2017; Damasceno et al., 2016; Rocca et al., 2014) but there were insufficient data for meta-analyses. In addition, studies reported microstructural changes within the basal ganglia, thalamus and frontal lobe and impaired functional connectivity between basal ganglia structures and the sensorimotor cortex, frontal, parietal and temporal lobes (Jaeger et al., 2018; Wilting et al., 2016). Impaired basal ganglia circuitry, including striatocortical and striatothalamic networks and potentially implicating regions that are heavily reliant on dopamine neurotransmission (e.g. ventral striatum), have been postulated to be important mechanistic factors underpinning perceived MS fatigue (Chaudhuri & Behan, 2000; Dobryakova et al., 2015). These regions are primarily responsible for motor control, motor planning, attentional control and the integration of afferent and efferent information.

### T1 and T2-Weighted Lesions

MS-HF showed an increased number of T1-weighted hypointense lesions in comparison with MS-LF, a difference that was even more pronounced in comparison with HC. However, there was no difference in the number of T2-weighted lesions or DTI indices of white matter microstructural integrity between MS-HF and MS-LF. The increased number of T1-weighted hypointense lesions could reflect associations between MS fatigue symptoms and recently activated immune inflammatory pathways or irreversible pathological changes which are features of the disease (Morris et al., 2016). Recently formed T1-weighted scan-identified hypointense lesions represent current disease activity, including reversible oedema, inflammation, demyelination and remyelination, whereas chronic T1-weighted hypointense lesions reflect irreversible demyelination and axonal loss (Napoli & Bakshi, 2005). In contrast, T2-weighted scan-identified lesions, which are non-specific for the underlying pathology, reflect the accumulated lesion load or “burden of disease” (Sinnecker et al., 2012) and occur throughout the brain and white matter, but less commonly, the grey matter (Napoli & Bakshi, 2005). Nevertheless, the possibility that localised white matter atrophy and loss of white matter microstructural integrity within specific brain regions could influence MS fatigue symptoms should not be overlooked. Consistent with this observation, there is evidence that atrophy progression within the corpus callosum (largest collection of brain white matter) is implicated in the evolution of MS-related fatigue (Yaldizli et al., 2010). Furthermore, other studies have provided evidence of localised metabolic alterations or anisotropic changes in white matter adjacent to the lateral and medial pre-frontal cortex and in fibres connecting basal ganglia structures (Hanken et al., 2015).

### Muscle Strength

Meta-analysis revealed a reduction in muscle strength (MVC) in MS-HF versus MS-LF, which consolidates a conflicting body of data on this measure from studies investigating PwMS (irrespective of fatigue status) versus HC (Zijdewind et al., 2016). Greater cortico-subcortical grey matter atrophy or structural damage in MS-HF versus MS-LF could have a more pronounced effect on neural transmission from the brain to active skeletal muscles, and this could account for the reduced MVC. Rocca et al. (2012) reported a more diffuse pattern of spinal cord interneuron activation in the axial and longitudinal planes in MS-HF versus MS-LF, which they speculated could be attributable to abnormally functioning local circuits, altered modulation from supraspinal pathways or local and remote structural damage (Rocca et al., 2012). However, findings from our meta-analyses suggest that the relative integrity of corticospinal motor pathways is similar in MS-HF and MS-LF, as there were no differences in MEP variables or central motor conduction time between the groups. In contrast, a significant increase in MEP threshold was apparent for MS-HF and MS-LF versus HC, consolidating inconsistent data from previous studies on this measure of corticospinal excitability (Zijdewind et al., 2016).

### Voluntary Activation

Our meta-analyses revealed clear evidence of impaired voluntary activation (central motor drive) in MS-HF versus MS-LF, suggesting MS-HF have a relatively impaired ability to fully activate skeletal muscles in comparison with MS-LF. This may explain the observed reduction in MVC in MS-HF versus MS-LF, as previous studies have reported significant correlations between the decline in MVC and voluntary activation during sustained muscular contractions in PwMS (Zijdewind et al., 2016). Although females are reported to record lower voluntary activation (and MVC) values than males (Solianik et al., 2017), the higher female to male ratio in the included studies is unlikely to account for these findings, as MS-HF and MS-LF comparison groups tended to be well-balanced for sex. An alternative explanation for the lower voluntary activation (and MVC) scores in MS-HF could be the deconditioning effects of relative physical inactivity after an MS diagnosis, which may be further compounded by the experience of severe MS fatigue (Sebastiao et al., 2017). Relative inactivity leads to disuse atrophy and neurophysiological changes affecting skeletal muscle voluntary activation, leading to impaired muscular strength and function (Rice et al., 1992). In turn, this could increase the amount of effort required for everyday tasks, thus exacerbating perceived fatigue and motor fatigability.

### Intracortical Inhibition and Facilitation

Current data provides no clear evidence of a link between MS fatigue and altered intracortical inhibition (SICI) or intracortical facilitation (ICF), despite reports of altered functional connectivity and hyperactivation in fronto-parietal cortical regions, sensorimotor network and subcortical areas important for motor, sensory and cognitive processing in MS-HF (Bisecco et al., 2017; Jaeger et al., 2018; Rocca et al., 2016; Specogna et al., 2012; Tartaglia et al., 2008). Evidence from fMRI and EEG studies suggests that functional reorganisation within cortico-subcortical networks as a compensatory response to MS brain lesions could account for an increased energy demand for neural processing within certain networks (Filippi & Rocca, 2004; Kos et al., 2008). This could at least partially explain increased perceptions of fatigue in PwMS because of an elevated demand on functioning neural circuits. However, at present very few studies have compared SICI or ICF variables between MS-HF and MS-LF, making it difficult to draw conclusions about the extent to which modulation of intracortical inhibitory or facilitatory networks could be implicated in MS fatigue. The limited conflicting data that is currently available for SICI may be a reflection of different MS populations studied, as two of the published studies were focused on people with relapsing–remitting MS (Liepert et al., 2005; Morgante et al., 2011), whereas a third was focused on progressive MS (Chalah et al., 2019).

### Upper- and Lower-Limb Motor Fatigability

Meta-analysis of five studies revealed an increased level of upper-limb motor fatigability for MS-HF versus MS-LF, showing a more pronounced decline in force production. In contrast, only one of the included studies with a small sample size (N = 9) compared lower-limb motor fatigability between MS-HF and MS-LF (Ng et al., 2000) using a sustained 30% MVC dorsi-flexor protocol. There is no standardised method for assessing motor fatigability (Severijns et al., 2017) and this is reflected in the broad range of protocols used in comparisons of PwMS versus HC. It is also acknowledged that motor fatigability is task specific, being influenced by task complexity (Wolkorte et al., 2015), and that heterogeneity between patients (attributable to MS-specific functional impairments and differences in motor control) can confound motor fatigability measures (Severijns et al., 2017). However, aside from measurement of force decline over time, consistent motor fatigability data for MS-HF versus MS-LF have been reported in studies that have used exercise duration and number of muscular contractions before reaching a fatigue criterion (Liepert et al., 2005; Perretti et al., 2004). Evidence suggests that motor fatigability resulting from a sustained voluntary muscle contraction in PwMS mainly results from a decline in voluntary activation (suggesting central fatigue mechanisms), whereas in healthy controls motor fatigability seems to be mainly of peripheral origin (peripheral fatigue mechanisms) at the level of skeletal muscle (Severijns et al., 2017; Sheean et al., 1997; Steens et al., 2012b). An elegant study that combined imaging and electrophysiological techniques showed that in PwMS there was an inability to increase cortical activation in response to motor fatigability-related changes downstream of the motor cortex, which was at odds with the increase in cortical activation observed in HC (Steens et al., 2012b). Our meta-analysis of upper-limb data suggests that MS-HF may have less ability than MS-LF to increase cortical activation as a compensatory response to peripheral fatigue and this warrants further study. In addition, the relative paucity of lower-limb studies needs to be addressed, as PwMS more commonly report issues of motor fatigability in relation to lower-limb activities such as walking (Severijns et al., 2017).

### Limitations

Key limitations of this meta-analysis include the diversity of MRI techniques used for neuroimaging studies and heterogeneity of methods used to assess self-reported perceived fatigue and motor fatigability. Furthermore, many studies collected either neuroimaging or neurophysiological data, which prevented an exploration of relationships between neuroanatomical and neurophysiological impairments (including the impact on motor fatigability measures) within the same participants. The broad-ranging patient characteristics and lack of participant ethnicity data across different studies may also be considered as a limitation, although confounders such as disease severity, level of disability, sex and age were minimised in the larger data-set meta-analyses. Nevertheless, some of the meta-analyses included a small number of studies and as the overall quality rating of included studies was ‘moderate’, as such, caution is needed when interpreting these results. In addition, although the Agency for Healthcare Research and Quality (Zeng et al., 2015) is suitable for use in systematic reviews of cross-sectional studies, there is no obvious candidate tool for assessing the quality of observational/cross-sectional studies, which may be considered a study limitation. Finally, the method used to differentiate MS-HF and MS-LF in the included studies was based on previously published cut-points for the FSS and MFIS that rely on recollections of fatigue experiences over the previous 1–4 weeks. Fatigue can be sporadic and the intensity of fatigue symptoms amongst PwMS at the time of testing was not well-documented in many studies. In addition, differences in the exact cut-points used to distinguish MS-HF from MS-LF across different studies have to be taken into account when interpreting meta-analysis data based on dichotomised categories.

## Conclusion

In conclusion, this is the first meta-analysis to synthesise published cross-sectional data on structural and neurophysiological measures between MS-HF and MS-LF. The results suggest that higher levels of MS fatigue are characterised by greater cortico-subcortical grey matter atrophy and brain lesions, reduced muscular strength, reduced central drive (voluntary activation), and increased upper-limb motor fatigability. By consolidating an extensive and somewhat conflicting evidence-base, the meta-analysis provides new insights into neurobiological differences that exist between MS-HF and MS-LF.

## Supplementary Information

Below is the link to the electronic supplementary material.

Supplementary file1 (DOCX 13982 KB)

Supplementary file2 (DOCX 9677 KB)

Supplementary file3 (DOCX 9680 KB)

Supplementary file4 (DOCX 15 KB)

Supplementary file5 (DOCX 38 KB)

Supplementary file6 (DOCX 215 KB)

## Data Availability

Data from the included primary studies is available in the supplementary tables and figures.
