# Supplementary material for: Neurostructural and Neurophysiological Correlates of Multiple Sclerosis Physical Fatigue: Systematic Review and Meta-Analysis of Cross-Sectional Studies
Source: Neuropsychol Rev. 2021 May 7;32(3):506–19. doi: 10.1007/s11065-021-09508-1 (PMC9381450; doi:10.1007/s11065-021-09508-1)
Supplement: Supplementary file 1 — Supplementary file1 (DOCX 13982 KB) [file 11065_2021_9508_MOESM1_ESM.docx]

**Supplementary Figure 1**. Forest plots for neuroimaging and neurofunctional variables (MS-HF versus MS-LF). Data are presented as absolute mean differences and 95% confidence intervals, with abscissas representing a reduction or increase in the variable of interest for MS-HF in comparison with MS-LF.

**Table 1.3** Regional brain volumes (ml)

**Table 1.1** Total brain volume (ml)

**Table 1.2** Brain parenchymal fraction (%)

**Table 1.4** Subcortical grey matter structure volumes (ml)

**Table 1.5** T1- and T2-weighted lesion volumes (ml)

**Table 1.6** Fractional anisotropy and mean diffusivity

**Table 1.7** NAA/Cr and Cho/Cr ratios

**Table 1.8** Maximum voluntary contraction (MVC) force (N)

**Table 1.9** Voluntary activation (%)

**Table 1.10** Motor evoked potential threshold (%)

**Table 1.11** Motor evoked potential amplitude (mV)

**Table 1.12** Motor evoked potential latency (ms)

**Table 1.13** Central motor conduction time (ms)

**Table 1.14** Short interval intracortical inhibition (%)

**Table 1.15** Intracortical facilitation (%)

**Table 1.16** Upper-limb fatigability: post-fatigue task maximum voluntary contraction (MVC) force (% baseline MVC)
