# Supplementary material for: Neurostructural and Neurophysiological Correlates of Multiple Sclerosis Physical Fatigue: Systematic Review and Meta-Analysis of Cross-Sectional Studies
Source: Neuropsychol Rev. 2021 May 7;32(3):506–19. doi: 10.1007/s11065-021-09508-1 (PMC9381450; doi:10.1007/s11065-021-09508-1)
Supplement: Supplementary file 2 — Supplementary file2 (DOCX 9677 KB) [file 11065_2021_9508_MOESM2_ESM.docx]

**Supplementary Figure 2**. Forest plots for neuroimaging and neurofunctional variables (MS-HF versus HC). Data are presented as absolute mean differences and 95% confidence intervals, with abscissas representing a reduction or increase in the variable of interest for MS-HF in comparison with HC.

**Table 2.1** Total brain volume (ml)

**Table 2.2** Brain parenchymal fraction (%)

**Table 2.3** Regional brain volumes (ml)

**Table 2.4** Subcortical grey matter structure volumes (ml)

**Table 2.5** T1-weighted lesion volume (ml)

**Table 2.6** Fractional anisotropy and mean diffusivity

**Table 2.7** NAA/Cr ratio

**Table 2.8** Maximum voluntary contraction (MVC) force (N)

**Table 2.9** Motor evoked potential threshold (%)

**Table 2.10** Motor evoked potential amplitude (mV)

**Table 2.11** Motor evoked potential latency (ms)

**Table 2.12** Short interval intracortical inhibition (%)

**Table 2.13** Intracortical facilitation (%)

**Table 2.14** Upper-limb fatigability: post-fatigue task maximum voluntary contraction (MVC) force (% baseline MVC)
