# Supplementary material for: Neurostructural and Neurophysiological Correlates of Multiple Sclerosis Physical Fatigue: Systematic Review and Meta-Analysis of Cross-Sectional Studies
Source: Neuropsychol Rev. 2021 May 7;32(3):506–19. doi: 10.1007/s11065-021-09508-1 (PMC9381450; doi:10.1007/s11065-021-09508-1)
Supplement: Supplementary file 3 — Supplementary file3 (DOCX 9680 KB) [file 11065_2021_9508_MOESM3_ESM.docx]

**Supplementary Figure 3**. Forest plots for neuroimaging and neurofunctional variables (MS-LF versus HC). Data are presented as absolute mean differences and 95% confidence intervals, with abscissas representing a reduction or increase in the variable of interest for MS-LF in comparison with HC.

**Table 3.1** Total brain volume (ml)

**Table 3.2** Brain parenchymal fraction (%)

**Table 3.3** Regional brain volumes (ml)

**Table 3.4** Subcortical grey matter structure volumes (ml)

**Table 3.5** T1-weighted lesion volume (ml)

**Table 3.6** Fractional anisotropy and mean diffusivity

**Table 3.7** NAA/Cr ratio

**Table 3.8** Maximum voluntary contraction (MVC) force (N)

**Table 3.9** Motor evoked potential threshold (%)

**Table 3.10** Motor evoked potential amplitude (mV)

**Table 3.11** Motor evoked potential latency (ms)

**Table 3.12** Short interval intracortical inhibition (%)

**Table 3.13** Intracortical facilitation (%)

**Table 3.14** Upper-limb fatigability: post-fatigue task maximum voluntary contraction (MVC) force (% baseline MVC)
