# Supplementary material for: Neurostructural and Neurophysiological Correlates of Multiple Sclerosis Physical Fatigue: Systematic Review and Meta-Analysis of Cross-Sectional Studies
Source: Neuropsychol Rev. 2021 May 7;32(3):506–19. doi: 10.1007/s11065-021-09508-1 (PMC9381450; doi:10.1007/s11065-021-09508-1)
Supplement: Supplementary file 4 — Supplementary file4 (DOCX 15 KB) [file 11065_2021_9508_MOESM4_ESM.docx]

**Supplementary Table 1.** Databases and search terms

| Database  Up to December 31^st^, 2019 | Search |
| --- | --- |
| PubMed/MEDLINE | (("multiple sclerosis"[MeSH Terms] OR ("multiple"[All Fields] AND "sclerosis"[All Fields]) OR "multiple sclerosis"[All Fields]) AND (twitch[All Fields] AND interpolation[All Fields])) AND central[All Fields] AND ("transcranial magnetic stimulation"[MeSH Terms] OR ("transcranial"[All Fields] AND "magnetic"[All Fields] AND "stimulation"[All Fields]) OR "transcranial magnetic stimulation"[All Fields] OR ("transcranial"[All Fields] AND "magnetic"[All Fields] AND "stimulation"[All Fields] AND "paired"[All Fields] AND "pulse"[All Fields]))) AND (intracortical[All Fields] AND excitability[All Fields]) AND (("nerve tissue"[MeSH Terms] OR ("nerve"[All Fields] AND "tissue"[All Fields]) OR "nerve tissue"[All Fields] OR "nerve"[All Fields]) AND stimulation[All Fields])) AND (maximal[All Fields] AND contraction[All Fields]) AND (motor[All Fields] AND execution[All Fields]) AND (maximal[All Fields] AND contraction[All Fields]) AND force[All Fields] AND (motor[All Fields] AND execution[All Fields]) AND motor fatigue AND force AND/OR (multiple sclerosis) AND motor fatigue AND fatigue scores AND ("neurophysiology"[MeSH Terms] OR "neurophysiology"[All Fields])) AND ("fatigue"[MeSH Terms] OR "fatigue"[All Fields]) AND muscle action potentials AND neural activity AND ("motor activity"[MeSH Terms] OR ("motor"[All Fields] AND "activity"[All Fields]) (10) "magnetic resonance imaging" MeSH Terms] OR ("magnetic"[All Fields] AND "resonance"[All Fields] AND "imaging"[All Fields]) OR "magnetic resonance imaging"[All Fields] OR "fmri"[All Fields]) "functional magnetic resonance imaging"[All Fields]) "functional"[All Fields] OR “volumetric” AND “brain structures” (("brain"[MeSH Terms] OR "brain"[All Fields]) AND structures[All Fields]) OR “brain function” (("brain"[MeSH Terms] OR "brain"[All Fields]) AND ("physiology"[Subheading] OR "physiology"[All Fields] OR "function"[All Fields] OR "physiology"[MeSH Terms] OR "function"[All Fields])) AND “brain activation "brain"[MeSH Terms] OR "brain"[All Fields]) AND activation [All Fields]. |
| ProQuest, | multiple sclerosis, nerve stimulation, twitch interpolation, transcranial magnetic stimulation, motor cortical excitability, muscle strength, peripheral and central fatigue, maximal voluntary contraction, motor task, maximal force, motor fatigue, fatigue scores, neurophysiology, muscle action potential, neural activity, magnetic resonance imaging, functional magnetic resonance imaging, volumetric, brain structures, brain function, brain activation. |
| CINAHL | multiple sclerosis, nerve stimulation, twitch interpolation, transcranial magnetic stimulation, motor cortical excitability, muscle strength, peripheral and central fatigue, maximal voluntary contraction, motor task, maximal force, motor fatigue, fatigue scores, neurophysiology, muscle action potential, neural activity, magnetic resonance imaging, functional magnetic resonance imaging, volumetric, brain structures, brain function, brain activation. |
| Web of Science | TS=('multiple sclerosis' AND 'transcranial magnetic stimulation, paired pulse')  TS=('multiple sclerosis' AND 'transcranial magnetic stimulation, single pulse')  TS=('multiple sclerosis' AND 'twitch interpolation')  TS=('multiple sclerosis' AND 'muscle strength')  TS=('multiple sclerosis' AND 'muscle fatigue, peripheral')  TS=('multiple sclerosis' AND 'muscle fatigue, central')  TS=('multiple sclerosis' AND 'motor performance')  TS=('multiple sclerosis' AND ‘nerve stimulation’)  TS=('multiple sclerosis' AND ‘brain stimulation’)  TS=('multiple sclerosis' AND ‘fatigue scales’)  TS=('multiple sclerosis' AND ‘magnetic brain imaging’)  TS=('multiple sclerosis' AND ‘functional magnetic brain imaging’)  TS=('multiple sclerosis' AND ‘brain function, brain structures’)  TS=('multiple sclerosis' AND ‘brain activation’)  TS=('multiple sclerosis' AND ‘neurophysiology’) |
