# Supplementary material for: Neurostructural and Neurophysiological Correlates of Multiple Sclerosis Physical Fatigue: Systematic Review and Meta-Analysis of Cross-Sectional Studies
Source: Neuropsychol Rev. 2021 May 7;32(3):506–19. doi: 10.1007/s11065-021-09508-1 (PMC9381450; doi:10.1007/s11065-021-09508-1)
Supplement: Supplementary file 5 — Supplementary file5 (DOCX 38 KB) [file 11065_2021_9508_MOESM5_ESM.docx]

**Supplementary Table 2.** Methodological quality of the included studies evaluated using the Cross-Sectional/Prevalence Study Quality Scale, recommended by the Agency for Healthcare Research and Quality (AHRQ): ⊕ reported; — not reported; U unclear; NA not applicable. Scores of 0-3 indicate “low quality”, 4–7 “moderate quality” and 8–11 “high quality”. Φ original data acquired from senior author of the publication; Ψ Studies not used for meta-analysis; Ω studies providing neuroimaging and neurofunctional data.

| Study | Define source of information (survey, record review) | List inclusion and exclusion criteria for exposed and unexposed subjects (cases and controls) or refer to previous publications | Indicate time period used for identifying patients | Indicate whether or not subjects were consecutive if not population-based | Indicate if evaluators of subjective components of study were masked to other aspects of the status of the participants | Describe any assessments undertaken for quality assurance purposes (e.g., test/retest of primary outcome measurements) | Explain any patient exclusions from analysis | Describe how confounding was assessed and/or controlled | If applicable, explain how missing data were handled in the analysis | Summarize patient response rates and completeness of data collection | Clarify what follow-up, if any, was expected and the percentage of patients for which incomplete data or follow-up was obtained | Total items positively reported | **Study quality rating** |
| --- | --- | --- | --- | --- | --- | --- | --- | --- | --- | --- | --- | --- | --- |
| Andreasen *et al*. (2009) | ⊕ | ⊕ | ⊕ | ⊕ | — | — | ⊕ | ⊕ | ⊕ | ⊕ | NA | 8 | **High** |
| Andreasen *et al*. (2010) Ω | ⊕ | ⊕ | ⊕ | — | — | — | ⊕ | ⊕ | — | ⊕ | NA | 6 | **Mod** |
| Bakshi *et al.* (1999) Ψ | ⊕ | ⊕ | ⊕ | ⊕ | ⊕ | — | ⊕ | — | — | ⊕ | NA | 7 | **Mod** |
| Bernitsas *et al*. (2017) | ⊕ | ⊕ | — | — | — | — | — | ⊕ | — | ⊕ | NA | 4 | **Mod** |
| Bisecco *et al*. (2016) | ⊕ | ⊕ | — | ⊕ | ⊕ | — | — | ⊕ | — | ⊕ | NA | 6 | **Mod** |
| Bisecco *et al*. (2017) Ψ | ⊕ | ⊕ | — | — | ⊕ | — | — | ⊕ | — | — | NA | 4 | **Mod** |
| Calabrese *et al*. (2010) | ⊕ | ⊕ | ⊕ | ⊕ | — | — | — | — | — | — | ⊕ | 5 | **Mod** |
| Chalah *et al.* (2019) Φ | ⊕ | ⊕ | — | — | — | — | ⊕ | — | — | ⊕ | NA | 4 | **Mod** |
| Codella *et al*. (2002) | ⊕ | ⊕ | — | — | — | — | — | ⊕ | — | — | NA | 3 | **Low** |
| Cogliati Dezza *et al*. (2015) Ω | ⊕ | ⊕ | — | — | — | — | — | ⊕ | — | — | NA | 3 | **Low** |
| Colombo *et al*. (2000) Ψ Ω | ⊕ | ⊕ | — | ⊕ | — | — | — | — | — | — | NA | 3 | **Low** |
| Conte *et al.* (2016) Ψ | ⊕ | ⊕ | ⊕ | — | ⊕ | — | ⊕ | — | ⊕ | ⊕ | NA | 7 | **Mod** |
| Cruz Gomez *et al*. (2013) | ⊕ | ⊕ | — | — | — | — | — | ⊕ | — | — | NA | 3 | **Low** |
| Damasceno *et al*. (2016) | ⊕ | ⊕ | — | — | ⊕ | — | — | ⊕ | — | ⊕ | NA | 5 | **Mod** |
| Derache *et al.* (2013) Ψ | ⊕ | ⊕ | ⊕ | — | — | — | — | ⊕ | — | ⊕ | NA | 5 | **Mod** |
| Dobryakova *et al*. (2018) Φ | ⊕ | ⊕ | — | — | — | — | ⊕ | ⊕ | ⊕ | — | NA | 5 | **Mod** |
| Filippi *et al.* (2002) Ψ | ⊕ | ⊕ | — | — | ⊕ | ⊕ | ⊕ | ⊕ | ⊕ | ⊕ | NA | 8 | **High** |
| Gobbi *et al*. (2014a) | ⊕ | ⊕ | — | ⊕ | — | — | — | ⊕ | — | ⊕ | NA | 5 | **Mod** |
| Gobbi *et al*. (2014b) Ψ | ⊕ | ⊕ | — | ⊕ | ⊕ | — | — | ⊕ | — | ⊕ | NA | 6 | **Mod** |
| Gonzalez Campo *et al*. (2019) Ψ | ⊕ | ⊕ | — | — | — | — | ⊕ | — | — | ⊕ | NA | 4 | **Mod** |
| Greim *et al.* (2007) | ⊕ | ⊕ | — | — | — | — | — | ⊕ | — | — | NA | 3 | **Low** |
| Hanken *et al*. (2015) | ⊕ | ⊕ | — | — | — | ⊕ | — | ⊕ | — | — | NA | 4 | **Mod** |
| Hanken *et al.* (2016) | ⊕ | ⊕ | — | — | — | — | — | ⊕ | — | ⊕ | NA | 4 | **Mod** |
| Hidalgo de la Cruz *et al*. (2017) | ⊕ | ⊕ | — | — | — | — | ⊕ | ⊕ | ⊕ | ⊕ | NA | 6 | **Mod** |
| Jaeger *et al*. (2018) | ⊕ | ⊕ | — | — | — | — | — | ⊕ | — | — | NA | 3 | **Low** |
| Leocani *et al*. (2001) Ψ | ⊕ | ⊕ | — | — | — | — | ⊕ | — | ⊕ | ⊕ | NA | 5 | **Mod** |
| Liepert *et al*. (2005) | ⊕ | ⊕ | — | — | — | — | — | — | — | — | NA | 2 | **Low** |
| Lin *et al*. (2019) Φ | ⊕ | ⊕ | — | — | — | — | ⊕ | ⊕ | ⊕ | — | NA | 5 | **Mod** |
| Morgante *et al*. (2011) Ω | ⊕ | ⊕ | ⊕ | — | — | — | ⊕ | ⊕ | ⊕ | ⊕ | NA | 7 | **Mod** |
| Ng *et al*. (2000) Φ | ⊕ | ⊕ | — | — | — | — | — | — | — | — | NA | 2 | **Low** |
| Ng *et al*. (2004) Φ | ⊕ | ⊕ | — | — | — | — | ⊕ | — | ⊕ | ⊕ | NA | 5 | **Mod** |
| Niepel *et al*. (2006) Ψ | ⊕ | ⊕ | ⊕ | — | ⊕ | ⊕ | ⊕ | ⊕ | — | — | NA | 7 | **Mod** |
| Pardini *et al.* (2010) | ⊕ | ⊕ | — | ⊕ | — | — | — | — | — | — | NA | 3 | **Low** |
| Pellicano *et al*. (2010) Φ | ⊕ | ⊕ | — | — | — | — | — | — | — | — | NA | 2 | **Low** |
| Perretti *et al.* (2004) | ⊕ | ⊕ | — | ⊕ | — | — | — | ⊕ | — | — | NA | 4 | **Mod** |
| Pravata *et al*. (2016) Ψ | ⊕ | ⊕ | — | — | — | — | ⊕ | ⊕ | ⊕ | ⊕ | NA | 6 | **Mod** |
| Riccitelli *et al*. (2011) | ⊕ | ⊕ | — | ⊕ | — | — | — | ⊕ | — | — | NA | 4 | **Mod** |
| Rocca *et al*. (2009) | ⊕ | ⊕ | — | ⊕ | — | — | — | ⊕ | — | — | NA | 4 | **Mod** |
| Rocca *et al*. (2012) Ψ | ⊕ | ⊕ | — | ⊕ | ⊕ | — | ⊕ | ⊕ | — | ⊕ | NA | 7 | **Mod** |
| Rocca *et al*. (2014) | ⊕ | ⊕ | — | ⊕ | — | — | — | ⊕ | — | U | NA | 4 | **Mod** |
| Rocca *et al*. (2016) | ⊕ | ⊕ | — | ⊕ | ⊕ | — | — | ⊕ | — | ⊕ | NA | 6 | **Mod** |
| Roelcke *et al*. (1997) Ψ | ⊕ | — | — | — | — | ⊕ | ⊕ | — | ⊕ | ⊕ | NA | 5 | **Mod** |
| Romani *et al.* (2004) Ψ | ⊕ | ⊕ | — | — | — | — | ⊕ | ⊕ | — | ⊕ | ⊕ | 6 | **Mod** |
| Russo *et al*. (2015) Ψ | ⊕ | ⊕ | — | — | ⊕ | — | ⊕ | — | ⊕ | ⊕ | NA | 6 | **Mod** |
| Sander *et al*. (2016) | ⊕ | ⊕ | ⊕ | — | — | ⊕ | ⊕ | ⊕ | ⊕ | ⊕ | NA | 8 | **High** |
| Scheidegger *et al*. (2012) Φ | ⊕ | ⊕ | — | — | — | — | — | U | — | — | NA | 2 | **Low** |
| Sebastiao *et al*. (2017) | ⊕ | ⊕ | — | — | — | — | — | ⊕ | — | ⊕ | NA | 4 | **Mod** |
| Severijns *et al*. (2019) Φ | ⊕ | ⊕ | — | — | — | — | — | ⊕ | — | — | NA | 3 | **Low** |
| Specogna *et al.* (2012) Ψ | ⊕ | ⊕ | — | — | ⊕ | — | ⊕ | — | — | ⊕ | NA | 5 | **Low** |
| Steens *et al*. (2012) Φ | ⊕ | ⊕ | — | — | — | — | — | — | — | — | NA | 2 | **Low** |
| Stefancin *et al.* (2019) | ⊕ | ⊕ | — | — | — | — | ⊕ | — | — | ⊕ | NA | 4 | **Mod** |
| Sepulcre *et al.* (2009) Φ Ψ | ⊕ | ⊕ | ⊕ | — | ⊕ | ⊕ | ⊕ | ⊕ | — | — | NA | 7 | **Mod** |
| Tartaglia *et al*. (2004) | ⊕ | ⊕ | ⊕ | — | — | — | ⊕ | ⊕ | ⊕ | — | NA | 6 | **Mod** |
| Tedeschi *et al*. (2007) | ⊕ | ⊕ | — | — | — | — | — | ⊕ | — | — | NA | 3 | **Low** |
| Tellez *et al*. (2008) | ⊕ | ⊕ | — | — | — | — | ⊕ | ⊕ | ⊕ | ⊕ | NA | 6 | **Mod** |
| Tomasevic *et al*. (2013) Ω | ⊕ | ⊕ | — | — | — | — | — | ⊕ | — | U | NA | 3 | **Low** |
| van der Werf *et al*. (1998) Ψ | ⊕ | ⊕ | — | — | ⊕ | — | ⊕ | — | — | ⊕ | NA | 5 | **Mod** |
| Vecchio *et al*. (2017) Ψ | ⊕ | ⊕ | — | — | — | — | — | ⊕ | — | — | NA | 3 | **Low** |
| Wilting *et al*. (2016) | ⊕ | — | — | — | — | — | ⊕ | — | ⊕ | ⊕ | NA | 4 | **Mod** |
| Wolkorte *et al*. (2015a) Φ | ⊕ | ⊕ | — | — | — | — | ⊕ | ⊕ | ⊕ | ⊕ | NA | 6 | **Mod** |
| Wolkorte *et al*. (2015b) Φ | ⊕ | ⊕ | ⊕ | — | — | — | ⊕ | ⊕ | ⊕ | ⊕ | NA | 7 | **Mod** |
| Wolkorte *et al*. (2016) Φ | ⊕ | ⊕ | — | — | — | — | ⊕ | ⊕ | ⊕ | ⊕ | NA | 6 | **Mod** |
| Yaldizli *et al*. (2011) | ⊕ | ⊕ | ⊕ | ⊕ | ⊕ | ⊕ | ⊕ | ⊕ | — | — | NA | 8 | **High** |
| Yarraguntla *et al*. (2019) | ⊕ | ⊕ | — | — | — | — | ⊕ | ⊕ | — | — | ⊕ | 5 | **Mod** |
| Zaini *et al*. (2016) Φ | ⊕ | ⊕ | — | — | — | — | ⊕ | U | ⊕ | U | NA | 4 | **Mod** |
| Zellini *et al.* (2009) Ψ | ⊕ | ⊕ | — | — | ⊕ | — | ⊕ | ⊕ | — | — | NA | 5 | **Mod** |
| **Items reported (%)** | 100 | 100 | 20 | 23 | 24 | 11 | 52 | 68 | 30 | 52 | 5 |  |  |
