# Supplementary material for: Neurostructural and Neurophysiological Correlates of Multiple Sclerosis Physical Fatigue: Systematic Review and Meta-Analysis of Cross-Sectional Studies
Source: Neuropsychol Rev. 2021 May 7;32(3):506–19. doi: 10.1007/s11065-021-09508-1 (PMC9381450; doi:10.1007/s11065-021-09508-1)
Supplement: Supplementary file 6 — Supplementary file6 (DOCX 215 KB) [file 11065_2021_9508_MOESM6_ESM.docx]

**Supplementary Table 3a.** Characteristics of neuroimaging studies included in this review (N=47).

| Author | N (by disease type) | MS Subgroups | Age (y) | Male/ Female | EDSS Scores | Disease  Duration (y) | Perceived Fatigue Measure | Technique | Outcomes | MS-HF | MS-LF | HC | Summary of findings |
| --- | --- | --- | --- | --- | --- | --- | --- | --- | --- | --- | --- | --- | --- |
| Andreason *et al*. (2010)** | 34 RR  7 HC | 17 MS-HF  17 MS-LF | MS-HF:  43 (27-53)  MS-LF:  39 (23-53)  HC:  39 (31-45) | MS-HF: 5/12  MS-LF: 9/8  HC: 1/6 | MS-HF: 3 (1-3.5)  MS-LF: 2 (1.5-3.5)  HC: 0 (0-2) | MS-HF: 5 (1-14)  MS-LF: 3 (0-9) | FSS  [>5 (Mean)]  MS-HF:  6.3 (5-7)  MS-LF:  2.8 (1-4)  HC:  2.7 (2-4) | MRI (DTI), MRS  3.0 Tesla scanner (GE Signa HDx) | Brain parenchymal fraction (%) | 81.5 ± 5.7 | 82.4 ± 2.5 | 81.1 ± 2.5 | Brain parenchymal fraction, lesion volume and NAA/Cr and DTI/MT indices were similar for MS-HF and MS-LF. Greater regional atrophy of grey matter structures and nearby white matter in MS-HF vs HC, found in frontal/parietal and basal ganglia regions. |
|  |  |  |  |  |  |  |  |  | Lesion load (%) | 0.53 ± 0.37 | 0.36 ± 0.28 |  |  |
|  |  |  |  |  |  |  |  |  | NAA/Cr | 1.29 ± 0.2 | 1.32 ± 0.19 | 1.38 ± 0.13 |  |
| Bakshi *et al.* (1999) | 66 MS | 46 MS-HF  20 MS-LF | MS-HF:  41.0 ± 1.4  MS-LF:  43.0 ± 4.0 | MS-HF: 14/32  MS-LF: 4/16 |  | MS: 9.7 (0.5-43) | FSS [>5.0 (mean)]  Means for the 2 groups not reported.  MS-HF≥5  MS-LF≤4 | MRI 1.0- or 1.5-Tesla | Brain atrophy; T1-hypointense and T2-hyperintense lesions. | Data only reported as correlation coefficients. | Data only reported as correlation coefficients. |  | No significant differences were found in any MRI measures between MS-HF and MS-LF groups. No significant correlation between fatigue and any of the regional or global MRI measures. |
| Bernitsas *et al.* (2017) | 29 RR | 15 MS-HF  14 MS-LF | MS-HF:  43 ± 2.9  MS-LF:  39 ± 1.7 |  | MS-HF: 2 (1-4)  MS-LF: 1.5 (1-4) | MS-HF: 10 ± 1.7  MS-LF: 8.6 ± 1.9 | FSS  [>5 (Mean)]  MS-HF:  6 ± 0.12  MS-LF:  1.89 ± 0.2 | MRI (DTI)  3.0 Tesla scanner (Siemens Verio) | T2 Lesion volume (ml) | 14.0 ± 9.7 | 15.3 ± 22.1 |  | Significantly lower subcortical grey matter volumes (thalamus, pallidus, and superior cerebellar peduncle) found in basal ganglia regions. FSS scores inversely correlated with thalamus and pallidus volumes. Lower fractional anisotropy and greater mean diffusivity in MS-HF vs MS-LF, demonstrating neuronal disruption. |
|  |  |  |  |  |  |  |  |  | Thalamus volume (ml) | 11.5 ± 1.1 | 14 ± 2.2 |  |  |
|  |  |  |  |  |  |  |  |  | Pallidus volume (ml) | 2.6 ± 0.27 | 3.0 ± 0.49 |  |  |
|  |  |  |  |  |  |  |  |  | Superior cerebellar peduncle volume (ml) | 207.3 ± 27.5 | 246.1 ± 35.9 |  |  |
|  |  |  |  |  |  |  |  |  | Fractional anisotropy (FA) | 0.24 ± 0.02 | 0.27 ± 0.03 |  |  |
|  |  |  |  |  |  |  |  |  | Mean diffusivity (×10^−3^ mm^2^/s) | 0.88 ± 0.08 | 0.82 ± 0.04 |  |  |
| Bisecco *et al*. (2016) | 60 RR  29 HC | 30 MS-HF  30 MS-LF | MS-HF:  41.2 (21-62)  MS-LF:  40.2 (23-54)  HC:  40.7 (25-61) | MS-HF: 10/20  MS-LF: 9/21  HC: 13/16 | MS-HF: 2.0 (1.0-6.0)  MS-LF: 1.5 (1-6.0) | MS-HF: 14.5 (1-44)  MS-LF: 11.5 (1-27) | FSS  [>4 (Mean)]  MS-HF:  5.2 (4.2-6.8)  MS-LF:  2 (1-3.6)  HC: 2 (1-3.9) | MRI (DTI)  3.0 Tesla scanner (Medical System) | Normalised brain volume (ml) | 428 ± 97 | 1444 ± 89 | 1513 ± 71 | Normalised brain volume, grey matter, white matter, T2 lesion volume were not significantly different between MS-HF vs MS-LF. MS-HF showed more extensive white matter damage than MS-LF for mean diffusivity and fractional anisotropy. |
|  |  |  |  |  |  |  |  |  | Grey matter volume (ml) | 777 ± 63 | 793 ± 61 | 824 ± 52 |  |
|  |  |  |  |  |  |  |  |  | White matter volume (ml) | 650 ± 43 | 651 ± 35 | 688 ± 35 |  |
|  |  |  |  |  |  |  |  |  | T2 Lesion volume (ml) | 10.1 ± 13.8 | 11.2 ± 12.1 |  |  |
| Bisecco *et al.* (2017) | 59 RR  29 HC | 28 MS-HF  31 MS-LF | MS:  40.1 ± 10.1  MS-HF:  40.5 (21-62)  MS-LF:  39.6 (23-54)  HC:  39.8 ± 10.6 | MS: 21/38  MS-HF: 10/18  MS-LF: 11/20  HC: 13/16 | MS: 2.0 (1.0-6.0)  MS-HF: 2.0 (1.0-5.5)  MS-LF: 1.5 (1-6.0) | MS: 12.5 (1-44)  MS-HF: 13.8 (1-44)  MS-LF: 11.2 (1-27) | FSS  [>4 (Mean)]  MS:  3.6 (1-6.4)  MS-HF: 5.1(4.1-6.4)  MS-LF:  2 (1-3.6)  HC:  1.9 (1-3.9) | MRI, fMRI  3.0 Tesla scanner (GE Medical) | T2 Lesion volume (ml) | 8.3 ± 12.9 | 11.2 ± 11.8 |  | Significantly increased functionality in posterior cingulate cortex but decreased in anterior cingulated cortex in MS-HF vs MS-LF and HC. Reorganisation found in both default mode network and sensorimotor network at rest. T2 lesion volume, normalised brain volume, grey matter and white matter volume were not significantly different between MS-HF vs MS-LF but were lower than HC. |
|  |  |  |  |  |  |  |  |  | Normalised brain volume (ml) | 1439 ± 96 | 1450 ± 89 | 1519 ± 80 |  |
|  |  |  |  |  |  |  |  |  | Grey matter volume (ml) | 784 ± 64 | 794 ± 61 | 830 ± 57 |  |
|  |  |  |  |  |  |  |  |  | White matter volume (ml) | 654 ± 40 | 656 ± 37 | 689 ± 37 |  |
| Calabrese *et al.* (2010) | 152 RR  42 HC | 71 MS-HF  81 MS-LF | MS-HF:  33.3 ± 8.2  MS-LF:  34.4 ± 8.8  HC:  35.5 ± 10.2 | MS-HF: 26/45  MS-LF: 29/52  HC: 16/26 | MS-HF: 3.3 ± 1.8  MS-LF: 2.2 ± 1.5 | MS-HF: 9.9 ± 7.1  MS-LF: 8.7 ± 1.5 | FSS  [≥4 (Mean)]  MS-HF:  5.1 ± 0.75  MS-LF:  2.2 ± 1.0 | MRI  1.5 Tesla scanner (Achieva MR) | Global mean cortical thickness (mm) | 2.17 ± 0.25 | 2.25 ± 0.21 | 2.50 ± 0.11 | Significant reductions in putamen, caudate and thalamus volumes and cortical thickness of the superior frontal gyrus and inferior parietal gyrus in MS-HF vs MS-LF and MS vs HC. T2 lesion volume was not significantly different for MS-HF vs MS-LF. |
|  |  |  |  |  |  |  |  |  | T2 lesion volume (ml) | 9.2 ± 7.9 | 8.8 ± 7.7 |  |  |
|  |  |  |  |  |  |  |  |  | Putamen (ml) | 3.8 ± 1.3 | 4.5 ± 0.8 | 5.1 ± 0.9 |  |
|  |  |  |  |  |  |  |  |  | Caudate (ml) | 4.1 ± 0.5 | 5.1 ± 0.5 | 5.2 ± 0.8 |  |
|  |  |  |  |  |  |  |  |  | Thalamus (ml) | 4.7 ± 0.9 | 6.8 ± 0.8 | 6.1 ± 0.8 |  |
|  |  |  |  |  |  |  |  |  | Superior frontal gyrus cortical thickness (mm) | 2.30 ± 0.28 | 2.57 ± 0.21 |  |  |
|  |  |  |  |  |  |  |  |  | Inferior parietal gyrus cortical thickness (mm) | 2.04 ± 0.28 | 2.52 ± 0.21 |  |  |
| Codella *et al.* (2002) | 28 MS  30 HC | 14 MS-HF  14 MS-LF | MS-HF:  39.1 ± 8.9  MS-LF:  37.6 ± 6.6  HC:  41.2 ± 7.1 | MS-HF: 3/11  MS-LF: 6/8  HC:10/20 | MS-HF: 1.0 (0.0-1.0)  MS-LF: 1.0 (0.0-1.0) | MS-HF: 6.0 (1-40)  MS-LF: 8.0 (3-22) | FSS  [>25 (Total)]  MS-HF:  38.9 (28-55)  MS-LF:  19.7 (13-24) | MRI (DTI, MTI)  1.5 Tesla scanner | T2 Lesion volume (ml) | 8.9 ± 10.8 | 7.2 ± 4.7 |  | T2 lesion volume and DTI indices were not significantly different for MS-HF vs MS-LF but the latter differed between MS and HC. |
|  |  |  |  |  |  |  |  |  | Mean diffusivity  (×10^−3^ mm^2^/s) | 0.93 ± 0.06 | 0.96 ± 0.04 | 0.91 ± 0.05 |  |
|  |  |  |  |  |  |  |  |  | Fractional anisotropy | 0.20 ± 0.01 | 0.20 ± 0.01 | 0.23 ± 0.01 |  |
|  |  |  |  |  |  |  |  |  | Magnetisation transfer ratio (%) | 40.0 ± 0.6 | 40.4 ± 1.2 | 40.6 ± 1.0 |  |
| Cogliati Dezza *et al.* (2015)** | 27 MS  8 HC | 15 MS-HF  12 MS-LF | MS-HF:  37.3 ± 4  MS-LF:  36.9 ± 7.5  HC:  37 (25-48) | MS-HF: 4/11  MS-LF:  4/8  HC: 1/7 | MS-HF: 1 (0-3)  MS-LF: 1 (0-2) | MS-HF: 3.9 ± 4.1  MS-LF:  7.1 ± 3.9 | MFIS  [>36 (Total)]  MS-HF:  42.1 ± 7.3  MS-LF:  19.9 ± 8.6 | MRI  1.5 Tesla scanner (Achieva, Phillips) | Thalamus volume (ml) | 14.1 ± 1.8 | 13.3 ± 1.5 | 14.7 ± 1.5 | Thalamus volume and rolandic thickness/asymmetry were not significantly different for MS-HF vs MS-LF or HC. |
|  |  |  |  |  |  |  |  |  | Intracranial volume (ml) | 1440 ± 200 | 1490 ± 230 | 1500 ± 210 |  |
|  |  |  |  |  |  |  |  |  | Central sulcus area (mm) |  |  |  |  |
|  |  |  |  |  |  |  |  |  | Left | 1.72 ± 0.07 | 1.72 ± 0.18 | 1.78 ± 0.14 |  |
|  |  |  |  |  |  |  |  |  | Right | 1.71 ± 0.10 | 1.68 ± 0.18 | 1.72 ± 0.15 |  |
| Colombo *et al.* (2000)** | 30 MS | 15 MS-HF  15 MS-LF | MS-HF:  30.4 (18-49)  MS-LF:  39 (18-49) | MS-HF: 3/12  MS-LF: 4/11 | MS-HF: 1.5 (0-1.5)  MS-LF: 1.5 (0-1.5) | MS-HF: 2.8 (1-7)  MS-LF: 3.7 (1-9) | FSS  [>25(Total)]  MS-HF:  40 (25-60)  MS-LF:  14 (10-21) | MRI  1.5 Tesla scanner | Total lesion load-volume | 32 (5-82) | 22 (6-60) |  | Significantly higher volume of lesions for MS-HF vs MS-LF, found in the parietal lobe and white matter regions (internal capsule, periventricular areas). |
| Cruz Gomez *et al.* (2013) | 60 RR  18 HC | 32 MS-HF  28 MS-LF | MS-HF:  37.72 ± 5.9  MS-LF:  34.96 ± 5.87  HC:  31.06 ± 5.67 | MS-HF: 11/21  MS-LF:  10/18  HC: 10/8 | MS-HF: 3.2 ± 1.68  MS-LF: 2.2 ± 0.96 |  | FSS  [>4 (Mean)]  MS-HF:  5.6 ± 0.85  MS-LF:  2.21 ± 0.96 | fMRI  1.5 Tesla scanner (Siemens Avanto) | T1 lesion volume (ml) | 6.03 ± 14.02 | 3.16 ± 3.97 |  | Grey matter and white matter atrophy in MS-HF vs MS-LF, found in areas related to the sensorimotor networks. Decreased resting functionality between the supplementary motor area and associative somato-sensory cortex in MS-HF vs MS-LF and HC and correlations with FSS scores. |
|  |  |  |  |  |  |  |  |  | Intracranial volume (ml) | 1101.16 ±  144.74 | 1141.34 ± 121.98 | 1261.24 ± 102.63 |  |
| Damasceno *et al.* (2016) | 49 RR  30 HC | 22 MS-HF  27 MS-LF | MS-HF:  31.86 ± 6.84 MS-LF:  30.18 ± 6.96  HC:  29.52 ± 7.53 | MS-HF: 3/19  MS-LF:  8/19  HC: 7/23 | MS-HF: 2.75 (1.5-4)  MS-LF:  1.5 (0-3.0) | MS-HF: 7.00 ± 4.82  MS-LF:  5.62 ± 4.79 | FSS  [>4 (Mean)]  MS:  3.54 ± 1.65  MS-HF:  5.19 ± 0.68  MS-LF:  2.20 ± 0.73  HC:  2.65 ± 0.88 | MRI  3.0 Tesla scanner (Achieva, Phillips) | Brain cortical grey matter volume (ml) | 426.20 ± 35.22 | 425.58 ± 51.88 | 440.82 ± 35.13 | Significantly greater cerebellar cortical lesion volume, brain cortical volume and most subcortical grey matter structures (thalamus, caudate, putamen, amygdala, accumbens) in MS-HF vs MS-LF and MS vs HC. Supports the theory of cortico-striatal network impairment in MS fatigue. |
|  |  |  |  |  |  |  |  |  | T1 lesion volume (ml) | 7.7 ± 12.0 | 4.7 ± 4.0 |  |  |
|  |  |  |  |  |  |  |  |  | Brain cortical lesions (mm^3^) | 900.0 ± 973.6 | 874.20 ± 870.0 |  |  |
|  |  |  |  |  |  |  |  |  | Cerebellar cortical lesion volume (mm^3^) | 91.56 ± 93.18 | 27.40 ± 56.29 | 94.36 ± 11.43 |  |
|  |  |  |  |  |  |  |  |  | Cerebellar grey matter volume (ml) | 88.87 ± 8.42 | 92.53 ± 16.96 | 94.36 ± 11.43 |  |
|  |  |  |  |  |  |  |  |  | Thalamus volume (ml) | 12.65 ± 1.42 | 12.06 ± 1.97 | 13.75 ± 1.18 |  |
|  |  |  |  |  |  |  |  |  | Putamen volume (ml) | 10.22 ± 2.10 | 10.82 ± 1.73 | 12.29 ± 1.40 |  |
|  |  |  |  |  |  |  |  |  | Caudate volume: (ml) | 6.47 ± 0.99 | 6.70 ± 1.05 | 7.56 ± 0.75 |  |
|  |  |  |  |  |  |  |  |  | Amygdala volume (ml) | 3.50 ± 0.61 | 3.66 ± 0.50 | 3.86 ± 0.55 |  |
|  |  |  |  |  |  |  |  |  | Accumbens volume (ml) | 1.28 ± 0.27 | 1.33 ± 0.26 | 1.55 ± 0.26 |  |
| Derache *et al.* (2013) | 17 RR | 11 MS-HF  6 MS-LF | MS-HF:  38 (20-49)  MS-LF:  26.5 (20-47) | MS-HF: 1/10  MS-LF:  0/6 | MS-HF:  2 (0-3)  MS-LF:  1.5 (1-2) | MS-HF:  3.9 (0.3-15.4)  MS-LF:  3.1 (0.6-4.1) | EMIF-SEP [≥45 (scaled)]  MS-HF:  56.9 (46.3-81.3)  MS-LF:  37.8 (12.2-43.1) | PET  (Seimens ECAT Exact HR+ scanner)  MRI 1.5 Tesla scanner (GE Inc. SIGNA Echo speed 8.3) | Regional grey matter density. Regional and global deep white matter lesion volume, regional and global juxtacortical and/or overlapping lesion volume. | Only difference scores between the groups and correlations reported. | Only difference scores between the groups and correlations reported. |  | Significant lower grey matter density in MS-HF versus MS-LF for the bilateral middle, superior and inferior frontal, left temporal and parietal cortex. Total fatigue score was negatively correlated with grey matter density in these same regions. |
| Dobryakova *et al.* (2018)^ | 17 RR  1 SP  1 PP  14 HC | 13 MS-HF  6 MS-LF | MS:  44.16 ± 7.46  HC:  37.29 ± 12.29 |  |  |  | FSS  [>36 (Total)]  MS-HF:  60 ± 3  MS-LF:  28 ± 8 | MRI, fMRI (+task)  3.0 Tesla | Grey matter volume (ml) | 756 ± 25 | 776 ± 54 | 810 ± 40 | Lower grey matter volume and white matter volume in MS-HF vs MS-LF and HC. |
|  |  |  |  |  |  |  |  |  | White matter volume (ml) | 692 ± 48 | 703 ± 58 | 739 ± 28 |  |
| Filippi *et al.* (2002) | 29 RR  15 HC | 15 MS-HF  14 MS-LF  15 HC | MS-HF:  39.3 ± 8.2  MS-LF:  37.6 ± 6.6 |  | MS-HF: 1 (0-1)  MS-LF:  1 (0-1) | MS-HF:  7 (1-40)  MS-LF:  6.5 (2-10) | FSS [≥25 (total)]  MS-HF:  39.5 ± 7.1  MS-LF:  19.3 ± 5.2 | MRI/fMRI  1.5 Tesla (Vision, Siemens) | Pattern of brain activation during a simple motor task (maximum finger-tapping frequency and the nine-hole peg test). | Data reported as brain scans and brain activation sites as Talairach coordinates. | Data reported as brain scans and brain activation sites as Talairach coordinates. | Data reported as brain scans and brain activation sites as Talairach coordinates. | Compared to MS-HF, MS-LF showed more significant activations of the ipsilateral cerebellar hemisphere, ipsilateral rolandic operculum, ipsilateral precuneus, contralateral thalamus and contralateral middle frontal gyrus during a simple motor task. In contrast, MS-HF had more significant activation of the contralateral cingulate motor area. Significant inverse correlations were found between FSS scores and relative activation of the contralateral intraparietal sulcus, ipsilateral rolandic operculum and thalamus. |
| Gobbi *et al.* (2014a) | 91 RR 22 SP 10 PP  90 HC | 81 MS-HF  66 MS-LF | MS-HF:  42.8 ± 11  MS-LF:  40.5 ± 10.5  HC:  41.9 ± 12.3 | MS-HF:  32/49  MS-LF:  28/38  HC: 33/57 | MS-HF: 3.5 (1.0-7.0)  MS-LF:  2.5 (0.0-7.0) | MS-HF: 13.3 ± 9.1  MS-LF:  12.1 ± 6.7 | FSS  [>4 (Mean)]  MS-HF:  5.2 (4-6.8)  MS-LF:  2.4 (1-3.9) | MRI (DTI)  3.0 Tesla scanner  (Intera, Philips Medical Systems) | Normalised brain volume (ml) | 1487 ± 106 | 1509 ± 107 | 1577 ± 85 | T1 and T2 lesion volume, normalised brain volume, grey matter and white matter volume did not significantly differ between MS-HF vs MS-LF but MS was lower vs HC. Reduced fractional anisotropy of the right anterior thalamic radiation and right uncinate fasciculus in MS-HF versus MS-LF. |
|  |  |  |  |  |  |  |  |  | Grey matter volume (ml) | 670 ± 76 | 685 ± 70 | 735 ± 50 |  |
|  |  |  |  |  |  |  |  |  | White matter volume (ml) | 817 ± 50 | 825 ± 61 | 841 ± 48 |  |
|  |  |  |  |  |  |  |  |  | T1 lesion volume (ml) | 6.4 ± 7.6 | 5.8 ± 5.9 |  |  |
|  |  |  |  |  |  |  |  |  | T2 lesion volume (ml) | 8.9 ± 10.0 | 8.4 ± 8.0 |  |  |
| Gobbi *et al.* (2014b) | 81 RR 18 BN 17 SP  8 PP  90 HC | 64 MS-HF  59 MS-LF | MS-HF:  42.3 ± 10.3  MS-LF:  41.0 ± 10.5  HC:  39.7 ± 13.7 | MS-HF: 26/38 MS-LF:  26/33  HC: 39/51 | MS-HF:  2.5 (1-7.0)  MS-LF:  1.5 (0.-7.0) | MS-HF: 13.1 (1-44)  MS-LF:  11.9 (1-32) | FSS  [>4 (Mean)]  MS-HF:  5.1 (3.0-6.6)  MS-LF:  2.3 (1.0-3.9) | MRI  3.0 Tesla scanner  (Intera, Philips Medical Systems) | Normalised brain volume (ml) | 1488 ± 107 | 1501 ± 112 | 1609 ± 86 | Normalised brain volume, T1 and T2 lesion volumes were not significantly different for MS-HF vs MS-LF. |
|  |  |  |  |  |  |  |  |  | T1 lesion volume (ml) | 6.8 ± 7.9 | 6.1 ± 6.1 |  |  |
|  |  |  |  |  |  |  |  |  | T2 lesion volume (ml) | 9.7 ± 10.7 | 8.8 ± 8.3 |  |  |
| Gonzalez Campo *et al.* (2019) | 27 RR  28 HC | 14 MS-HF  13 MS-LF | MS-HF:  42.2 ± 11  MS-LF:  36.3 ± 9.4  HC:  35.8 ± 11.0 | MS-HF: 5/14  MS-LF: 4/9  HC: 5/23 | MS-HF: 1.4 ±1.9  MS-LF: 1.0 ± 1.4 | MS-HF: 11.3 ±9.3  MS-LF: 7.9 ± 4.9 | MFIS  [≥37 (Total)]  MS-HF:  53.2 (37-76)  MS-LF:  19.9 (4-32) | MRI  1.5 T Phillips Intera scanner with a standard head coil | Introception condition accuracy score | –0.49 ± 0.83 | 0.64 ± 0.99 | 0.55 ± 0.87 | Decreased introception condition accuracy score and reduced grey matter voume in introceptive areas (bilateral insula, right anterior cingulate cortex) in MS-HF versus HC. Increased connectivity between the right anterior cingulate cortex and left insula in MS-HF versus HC. |
| Hanken *et al*. (2015) | 49 RR  17 HC | 28 MS-HF  25 MS-LF | MS-HF:  41.9 ± 6.8  MS-LF:  44.5 ± 11.2  HC:  37.4 ± 9.9 | MS-HF: 4/24  MS-LF:  6/19  HC: 6/11 | MS-HF: 2.9 ± 2.0  MS-LF:  2.8 ± 1.7 | MS-HF: 7.8 ± 8.3  MS-LF: 7.8 ± 6.1 | FSS  [>36 (Total)]  MS-HF:  54.6 ± 5.1  MS-LF:  29.8 ± 9.3  HC:  24.3 ± 7.8 | MRI (DTI)  3.0 Tesla scanner (Siemens Verio) | Brain parenchymal fraction (%) | 79.3 ± 3.6 | 78.7 ± 3.2 | 81.4 ± 3.0 | Brain parenchymal fraction, lateral, third and fourth ventricle volumes were not significantly different for MS-HF vs MS-LF or HC. |
|  |  |  |  |  |  |  |  |  | Lateral ventricles (ml) | 26.6 ± 15.5 | 28.2 ± 16.0 | 17.3 ± 6.9 |  |
|  |  |  |  |  |  |  |  |  | Third ventricle (ml) | 2.6 ± 1.7 | 2.6 ± 1.1 | 2.0 ± 1.0 |  |
|  |  |  |  |  |  |  |  |  | Fourth ventricle (ml) | 2.1 ± 0.9 | 2.0 ± 0.8 | 1.9 ± 0.8 |  |
| Hanken *et al.* (2016) | 69 RR  17 SP  9 PP  15 HC | 18 MS-HF  42 MS-LF | MS-HF:  43.1 ± 6.3  MS-LF:  42.9 ± 11.7  HC:  37.0 ± 10.5 | MS-HF: 4/14  MS-LF: 18/24  HC: 6/9 | MS-HF: 3.4 ± 2.2  MS-LF: 3.1 ± 1.8 | MS-HF:  6.8 ± 6.0  MS-LF:  7.8 ± 8.6 | FSS  [>5 (Mean)]  MS-HF:  56 ± 3  MS-LF:  30 ± 12 | MRI  1.5 Tesla scanner (Phillips) | Brain parenchymal fraction (%) | 80.8 ± 4.8 | 81.0 ± 4.3 | 81.8 ± 2.6 | Significantly decreased cortical thickness in right inferior parietal lobe, right cingulate cortex and the right precuneus for MS-HF vs MS-LF and HC. |
|  |  |  |  |  |  |  |  |  | Cortical thickness by region (mm): |  |  |  |  |
|  |  |  |  |  |  |  |  |  | Gyrus rectus Left | 3.0 ± 0.4 | 3.2 ± 0.3 | 3.3 ± 0.2 |  |
|  |  |  |  |  |  |  |  |  | Olfactory cortex left | 2.9 ± 0.3 | 2.9 ± 0.3 | 3.1 ± 0.1 |  |
|  |  |  |  |  |  |  |  |  | Inferior parietal right | 3.0 ± 0.4 | 3.2 ± 0.2 | 3.1 ± 0.1 |  |
|  |  |  |  |  |  |  |  |  | Precuneus right | 3.0 ± 0.3 | 3.1 ± 0.2 | 3.2 ± 0.1 |  |
|  |  |  |  |  |  |  |  |  | Superior temporal pole right | 3.5 ± 0.4 | 3.6 ± 0.3 | 3.8 ± 0.2 |  |
|  |  |  |  |  |  |  |  |  | Medial temporal pole right | 3.4 ± 0.4 | 3.6 ± 0.4 | 3.8 ± 0.3 |  |
|  |  |  |  |  |  |  |  |  | Anterior cingulate right | 2.9 ± 0.4 | 3.0 ± 0.2 | 3.0 ± 0.2 |  |
|  |  |  |  |  |  |  |  |  | Middle cingulate left | 2.9 ± 0.4 | 2.9 ± 0.3 | 3.1 ± 0.2 |  |
|  |  |  |  |  |  |  |  |  | Middle cingulate right | 2.9 ± 0.4 | 3.0 ± 0.2 | 3.1 ± 0.2 |  |
|  |  |  |  |  |  |  |  |  | Insula left | 2.7 ± 0.3 | 2.7 ± 0.3 | 2.9 ± 0.1 |  |
|  |  |  |  |  |  |  |  |  | Insula right | 2.7 ± 0.3 | 2.7 ± 0.2 | 2.9 ± 0.2 |  |
|  |  |  |  |  |  |  |  |  | Parahippocampus right | 2.9 ± 0.3 | 2.9 ± 0.4 | 3.0 ± 0.2 |  |
| Hidalgo de la Cruz *et al.* (2017) | 122 MS  94 HC | 36 MS-HF  86 MS-LF | MS-HF:  44.3 ± 12.4  MS-LF:  35.0 ± 11.8  HC:  41.5 ± 14.6 | MS-HF: 13/23  MS-LF: 37/49  HC: 46/48 | MS-HF: 4.0 (0.0-6.5)  MS-LF: 1.5 (0.0-8.0) | MS-HF: 13.4 ± 9.8  MS-LF: 10.8 ± 6.2 | MFIS  [>38 (Total)]  MS-HF: 48.0 (38.0-70.0)  MS-LF: 20.0 (0.0-37.0) | MRI (DTI), fMRI  3.0 Tesla scanner | Normalised brain volume (ml) | 1502 ± 102 | 1545 ± 86 | 1577 ± 84 | Significantly higher T1 and T2 lesion volumes and lower normalised brain volume and grey matter volume in MS-HF vs MS-LF. Resting state functional connectivity was not significantly different for MS-HF vs MS-LF or HC. |
|  |  |  |  |  |  |  |  |  | Grey matter volume (ml) | 682 ± 74 | 720 ± 58 | 736 ± 57 |  |
|  |  |  |  |  |  |  |  |  | White matter volume (ml) | 819 ± 42 | 826 ± 46 | 841 ± 43 |  |
|  |  |  |  |  |  |  |  |  | T1 lesion volume (ml) | 6.0 ± 6.5 | 3.5 ± 3.5 |  |  |
|  |  |  |  |  |  |  |  |  | T2 lesion volume (ml) | 9.0 ± 8.3 | 5.7 ± 5.3 |  |  |
|  |  |  |  |  |  |  |  |  | nRThal volume (ml) | 9.6 ± 1.2 | 9.8 ± 0.9 | 10.3 ± 0.8 |  |
|  |  |  |  |  |  |  |  |  | nLThal volume (ml) | 9.8 ± 1.0 | 10.1 ± 0.9 | 10.7 ± 0.8 |  |
| Jaeger *et al.* (2018) | 77 RR  41 HC | 39 MS-HF  38 MS-LF | MS-HF:  40 (18)  MS-LF:  34.5 (18)  HC:  36 (21) | MS-HF: 7/32  MS-LF: 14/24  HC: 15/26 | MS-HF: 2.5 (1)  MS-LF: 2 (1.5) | MS-HF: 6.8 (10.3)  MS-LF: 5.1 (9.1) | FSS  [>4 (Mean)]  MS-HF:  5.2 (1.3)  MS-LF:  2.6 (1.9)  HC:  1.9 (1.3) | MRI, fMRI  3.0 Tesla (Siemens Tim Trio scanner) | Total T1 white matter lesion volume (ml) | 3.7 (2.9) | 3.6 (4.2) |  | Significantly reduced functional connectivity of the whole caudate nucleus with sensorimotor and frontal, parietal, and temporal cortex regions in MS-HF vs MS-LF and HC. T1 lesion volume, normal brain volume, grey matter volume and white matter volume was not significantly different for MS-HF vs MS-LF but was lower for MS-HF than HC. |
|  |  |  |  |  |  |  |  |  | Normalised brain volume (ml) | 1522.4 ± 65.5 | 1548 ± 88.4 | 1565.5 ± 82.3 |  |
|  |  |  |  |  |  |  |  |  | Grey matter volume (ml) | 804.2 ± 57.2 | 824.2 ± 63.8 | 829 ± 63.6 |  |
|  |  |  |  |  |  |  |  |  | White matter volume (ml) | 718.2 ± 41.2 | 723.8 ± 38.1 | 736.5 ± 40.6 |  |
|  |  |  |  |  |  |  |  |  | Caudate volume (ml) | 9.3 ± 1.0 | 9.5 ± 0.78 | 9.7 ± 1.08 |  |
|  |  |  |  |  |  |  |  |  | Putamen volume (ml) | 12.5 ± 0.9 | 12.7 ± 0.9 | 13.3 ± 0.9 |  |
| Lin *et al.* (2019)^ | 34 RR  14 SP  4 PP  6 RSP  26 HC | 33 MS-HF  25 MS-LF | MS-HF:  53 ± 9.4  MS-LF:  53.6 ± 11.1  HC:  49.85± 14.4 | MS-HF: 8/25  MS-LF: 14/11  HC: 8/18 | MS-HF: 4.0 (2.5-6.5)  MS-LF: 2.5 (1.5-3.5) | MS-HF: 24 ± 11.3  MS-LF: 18.8 ± 8.8 | FSS  [>5 (Mean)]  MS-HF:  5.7 ± 0.9  MS-LF:  2.6 ± 0.9  HC:  2.59 ± 1.26 | MRI, fMRI  3.0 Tesla  (GE Signa Excite HD 12.0 8-channel scanner) | T2 lesion volume (ml) | 18.8 ± 22.0 | 12.0 ± 14.5 | 0.48 ± 1.23 | Decreased functional connectivity for MS vs HC between the left medial thalamic nuclei and left angular gyrus and reduced functional connectivity between the left posterior thalamic nuclei and left supramarginal gyrus, as well as decreased right medial thalamic nuclei connectivity with bilateral caudate/thalamus and left cerebellar areas. MS also had increased FC between the left anterior thalamic nuclei and anterior cingulate cortex bilaterally. Data for MS-HF and MS-LF obtained by communication with the author. |
|  |  |  |  |  |  |  |  |  | Intracranial volume (ml) | 1439.8 ± 81.1 | 1419.4 ± 66.5 | 1457.8 ± 143.5 |  |
|  |  |  |  |  |  |  |  |  | Thalamus volume (ml) | 13.2 ± 1.2 | 13.4 ± 1.5 | 15.1 ± 1.3 |  |
| Morgante *et al.* (2011)** | 33 RR  12 HC | 16 MS-HF  17 MS-LF | MS-HF:  41.1 ± 10.9  MS-LF:  38 ± 9.4 | MS-HF: 7/9  MS-LF: 4/13 | MS-HF: 1.8 ± 0.6  MS-LF: 1.6 ± 0.6 | MS-HF: 8.4 ± 3.4  MS-LF: 7.9 ± 3.8 | FSS  [>4 (Mean)]  MS-HF:  4.9 ± 0.8  MS-LF:  2.2 ± 0.9 | MRI  1.5 Tesla scanner (Magnetom Impact) | Total brain volume (ml) | 983.8 ± 102.8 | 995.7 ± 72.6 |  | Significantly higher T1 lesion volume in MS-HF vs MS-LF. Total brain volume, grey matter volume, white matter volume and T1 and T2 lesion volumes were not significantly different for MS-HF vs MS-LF. |
|  |  |  |  |  |  |  |  |  | Grey matter volume (ml) | 425.6 ± 64.4 | 447.1 ± 50.7 |  |  |
|  |  |  |  |  |  |  |  |  | White matter volume (ml) | 558.2 ± 84.4 | 548.6 ± 58.5 |  |  |
|  |  |  |  |  |  |  |  |  | T1 lesion volume (ml) | 1.3 ± 1.6 | 0.8 ± 1.0 |  |  |
|  |  |  |  |  |  |  |  |  | T2 lesion volume (ml) | 4.3 ± 5.0 | 3.2 ± 3.4 |  |  |
| Niepel *et al.* (2006) | 34 RR  19 HC | 20 MS-HF  11 MS-LF | MS:  38 (32-42) | MS: 7/27 | MS: 2.5 (2-3) | MS: 9 (3-13) | FSS  [≥5 (Mean)]  MS-HF≥5  MS-LF≤4  Means for the 2 MS groups not reported. | MRI  1.5 Tesla scanner (Vision MT) | T2 lesion volume (ml) | 5.9 (2.9-12.7) | 4.2 (2.0-7.4) |  | Median T2 lesion volume was not significantly different for MS-HF vs MS-LF. |
| Pardini *et al.* (2010) | 40 RR | 15 MS-HF  25 MS-LF | MS-HF:  41.3 ± 4.4  MS-LF:  36.0 ± 9.0 | MS:12/28 | MS-HF: 1.6 ± 1.2  MS-LF:  1.5 ± 0.7 | MS-HF: 5.9 ± 7.3  MS-LF:  5.8 ± 3.9 | MFIS  [>38 (Total)]  MS: 31.1 ± 18.0  MS-HF:  20.2 ± 10.0  MS-LF:  51.4 ± 9.9 | MRI (DTI)  1.5 Tesla scanner (MR system) | T2 Lesion load-volume (ml) | 8.6 ± 16.8 | 7.4 ± 9.5 |  | Significant involvement of different frontal (fronto-frontal, fronto-straital, fronto-occipital and fronto-limbic) networks in the pathophysiology of MS fatigue. Significant correlation between MFIS and white matter regions, within fronto-striatal networks. |
|  |  |  |  |  |  |  |  |  | Normalised Brain Volume (ml) | 1600 ± 900 | 1500 ± 200 |  |  |
| Pellicano *et al.* (2010)^ | 20 RR  4 SP  24 HC | 8 MS-HF  16 MS-LF | MS:  45.4 ± 9.7  HC:  45.1 ± 11.1 | MS: 7/17  HC: 7/17 | MS: 1.5 (0.0-6.5) | MS:  12.6 ± 8.4 | MFIS  [>38 (Total)]  MS:  30.3 ± 16.1  HC:  13.4 ± 12.1 | MRI  3.0 Tesla scanner  (Sigma MI) | T2 Lesion volume (ml) | 10.33 ± 9.8 | 8.60 ± 6.3 |  | T2 lesion volume was significantly higher and thickness of the posterior and inferior parietal cortex, supramarginal gyrus and thalamus volume lower for MS-HF vs MS-LF. |
| Pravata *et al.* (2016) | 22 RR  12 HC | 11 MS-HF  11 MS-LF  12 HC | MS-HF:  46.6 ± 9.3  MS-LF:  40.0 ± 5.8  HC: 41.4 ± 8.0 | MS-HF:  7/4  MS-LF:7/4  HC: 6/6 | MS-HF: 2.5 (0-3.5)  MS-LF:  1.5 (0-3.0) | MS-HF:  9.5 ± 3.8  MS-LF:  6.0 ± 4.4 | FSMC [≥22 (Cognitive Scale)]  MS-HF:  33.6 ± 4.8  MS-LF:  14.3 ± 3.8  HC:  14.4 ± 4.1 | MRI/fMRI  (3.0 Tesla Siemens “Skyra” scanner) | Brain resting-state functional connectivity (RS-FC) scans before, immediately after and 30 min after execution of the paced auditory serial addition test (PASAT). | Data reported as graphs and correlation coefficients. | Data reported as graphs and correlation coefficients. | Data reported as graphs and correlation coefficients. | MS-HF experienced stronger RS-FC 30 min post-PASAT between the left superior frontal gyrus and occipital, frontal and temporal areas. Also, in MS-HF, the left superior frontal gyrus was hyperconnected with the left caudate nucleus immediately post task and hypoconnected at 30 min post with the left anterior thalamus. |
| Riccitelli *et al.* (2011) | 24 RR  14 HC | 10 MS-HF  14 MS-LF | MS-HF:  38.0 ± 7.7  MS-LF:  38.6 ± 8.5  HC:  38.7 ± 8.4 | MS-HF: 6/4  MS-LF: 6/8  HC:  6/8 | MS-HF: 1.5 (1.5-2.0)  MS-LF: 1.5 (0-1.5) | MS-HF: 8.2 ± 6.2  MS-LF: 10.6 ± 6.6 | FSS  [>4 (Mean)]  MS-HF:  4.4 (4-6.1)  MS-LF: 2.1 (1.4-3.3) | MRI  1.5 Tesla scanner (Vision) | Total brain volume (ml) | 1596 ± 79 | 1560 ± 51 | 1649 ± 48 | Significantly greater atrophy of left central sulcus, precentral gyrus and primary motor cortex region in MS-HF vs MS-LF and HC. T2 lesion volume, total brain volume, white matter volume, grey matter volume and intracranial volume were not significantly different for MS-HF vs MS-LF but were lower compared to HC. |
|  |  |  |  |  |  |  |  |  | Grey matter volume (ml) | 795 ± 66 | 770 ± 45 | 826 ± 58 |  |
|  |  |  |  |  |  |  |  |  | White matter volume (ml) | 801 ± 55 | 790 ± 60 | 822 ± 42 |  |
|  |  |  |  |  |  |  |  |  | T2 Lesion load-volume (ml) | 7.7 ± 5.0 | 8.9 ± 10.7 |  |  |
| Rocca *et al.* (2009) | 24 RR  14 HC | 11 MS-HF  13 MS-LF | MS-HF:  33.8 ± 6.2  MS-LF:  31.2 ± 4.3  HC:  32.2 ± 5.5 | MS-HF: 1/10  MS-LF: 1/12  HC: 2/12 | MS-HF: 1.0 (0.0-1.5)  MS-LF: 1.0 (0.0-1.5) | MS-HF:  6 (2-12)  MS-LF:  6 (2-10) | FSS  [≥25 (Total)]  MS-HF:  37.7 ± 7.7  MS-LF:  15.6 ± 4.8 | MRI, fMRI  1.5 Tesla scanner (Vision Siemens) | Normalised brain volume (ml) | 1493 ± 131 | 1540 ± 100 | 1519 ± 115 | T2 lesion volume and normalised brain volume were similar for MS-HF and MS-LF and for MS vs HC. fMRI disruption in frontal-parietal lobes and basal ganglia regions in MS-HF vs MS-LF. |
|  |  |  |  |  |  |  |  |  | T2 lesion volume (ml) | 8.1 ± 8.5 | 8.8 ± 6.0 |  |  |
| Rocca *et al.* (2012) | 35 RR  20 HC | 20 MS-HF  15 MS-LF | MS-HF:  38.8 (29-65)  MS-LF:  38.8 (29-60)  HC:  37.3 (24-53) | MS-HF: 6/14  MS-LF: 8/7  HC: 7/13 | MS-HF: 3.5 (1.5-5.5)  MS-LF: 3.0 (0-5.0) | MS-HF: 10.5 (2-23)  MS-LF: 11.2 (0.5-27) | FSS  [>4 (Mean)]  MS-HF:  4.7 (4-6)  MS-LF:  2.2 (1-3.9) | MRI (DTI), fMRI  1.5 Tesla scanner (Siemens Magnetom) | T2 lesion volume (ml) | 6.6 ± 6.1 | 22.1 ± 16.5 |  | Significantly greater brain white matter fractional anisotropy and reduced fMRI activation in the left anterior and posterior cervical cord quadrants in MS-HF vs MS-LF and HC. |
|  |  |  |  |  |  |  |  |  | Mean diffusivity (×10^−3^ mm^2^/s) |  |  |  |  |
|  |  |  |  |  |  |  |  |  | Grey matter average | 0.94 ± 0.06 | 0.97 ± 0.07 | 0.89 ± 0.03 |  |
|  |  |  |  |  |  |  |  |  | White matter average | 0.79 ± 0.02 | 0.82 ± 0.06 | 0.77 ± 0.02 |  |
|  |  |  |  |  |  |  |  |  | Cervical cord average | 0.94 ± 0.09 | 0.96 ± 0.04 | 0.85 ± 0.09 |  |
|  |  |  |  |  |  |  |  |  | Fractional anisotropy (FA) |  |  |  |  |
|  |  |  |  |  |  |  |  |  | White matter average | 0.39 ± 0.02 | 0.36 ± 0.03 | 0.41 ± 0.02 |  |
|  |  |  |  |  |  |  |  |  | Cervical cord average | 0.47 ± 0.04 | 0.49 ± 0.04 | 0.59 ± 0.06 |  |
| Rocca *et al.* (2014) | 63 RR  35 HC | 31 MS-HF  32 MS-LF | MS-HF:  41 (23-63)  MS-LF:  39.5 (27-58)  HC:  40.7 (23-63) | MS-HF: 14/17  MS-LF: 15/17  HC: 16/19 | MS-HF: 2.5 (1.0-5.5)  MS-LF: 2.0 (1.0-6.5) | MS-HF: 13.0 (0.6-32)  MS-LF: 11.65 (0.8-25.5) | FSS  [>4 (Mean)]  MS-HF:  5.0 (4-6.4)  MS-LF:  2.1 (1.2-3.8) | MRI (DTI)  3.0 Tesla scanner  (Intera; Philips Medical Systems) | Normalised brain Volume (ml) | 1477 ± 109 | 1525 ± 90 | 1572 ± 93 | Significantly greater T2 lesion volume and atrophy of the right accumbens, right inferior temporal gyrus, left superior frontal gyrus, and forceps major in MS-HF vs MS-LF and HC. Lower fractional anisotropy (forceps major, left inferior fronto occipital fasciculus and right anterior thalamic radiation) in MS-HF vs MS-LF and HC. T1 lesion volume, normal brain volume, grey matter volume and white matter volume were not significantly different for MS-HF vs MS-LF but were lower compared to HC. |
|  |  |  |  |  |  |  |  |  | Grey matter Volume (ml) | 665 ± 85 | 691 ± 60 | 726 ± 58 |  |
|  |  |  |  |  |  |  |  |  | White matter Volume (ml) | 811 ± 46 | 835 ± 61 | 846 ± 52 |  |
|  |  |  |  |  |  |  |  |  | T1 lesion volume (ml) | 6.0 ± 6.1 | 5.7 ± 5.6 | 0.02 ± 0.04 |  |
|  |  |  |  |  |  |  |  |  | T2 lesion volume (ml) | 8.7 ± 9.7 | 8.5 ± 8.1 | 0.03 ± 0.09 |  |
|  |  |  |  |  |  |  |  |  | Mean cortical lesions (ml) | 2 (0-6) | 1 (0-8) |  |  |
|  |  |  |  |  |  |  |  |  | Thalamus  volume (ml) | 14.03 ± 1.13 | 14.27 ± 1.27 | 15.90 ± 1.13 |  |
|  |  |  |  |  |  |  |  |  | Caudate volume (ml) | 6.12 ± 0.64 | 6.43 ± 0.71 | 6.77 ± 0.57 |  |
|  |  |  |  |  |  |  |  |  | Putamen volume (ml) | 8.48 ± 0.71 | 8.69 ± 0.92 | 9.18 ± 0.71 |  |
|  |  |  |  |  |  |  |  |  | Accumbens volume (ml) | 0.75 ± 0.13 | 0.85 ± 0.14 | 0.85 ± 0.13 |  |
|  |  |  |  |  |  |  |  |  | Amygdala volume (ml) | 2.70 ± 0.22 | 2.67 ± 0.22 | 2.59 ± 0.22 |  |
|  |  |  |  |  |  |  |  |  | Hippocampus volumen (ml) | 6.87 ± 0.57 | 7.05 ± 0.64 | 7.30 ± 0.50 |  |
|  |  |  |  |  |  |  |  |  | Pallidus volumen (ml) | 3.16 ± 0.28 | 3.28 ± 0.28 | 3.26 ± 0.28 |  |
|  |  |  |  |  |  |  |  |  | Mean diffusivity (×10^−3^ mm^2^/s): |  |  |  |  |
|  |  |  |  |  |  |  |  |  | Grey matter | 0.92 ± 0.05 | 0.92 ± 0.05 | 0.90 ± 0.04 |  |
|  |  |  |  |  |  |  |  |  | White matter | 0.78 ± 0.03 | 0.78 ± 0.03 | 0.76 ± 0.02 |  |
|  |  |  |  |  |  |  |  |  | Fractional anisotropy: |  |  |  |  |
|  |  |  |  |  |  |  |  |  | Grey matter | 0.16 ± 0.01 | 0.16 ± 0.01 | 0.15 ± 0.01 |  |
|  |  |  |  |  |  |  |  |  | White matter | 0.38 ± 0.02 | 0.38 ± 0.02 | 0.39 ± 0.02 |  |
| Rocca *et al.* (2016) | 79 RR  26 HC | 50 MS-HF  29 MS-LF | MS-HF:  42.6 ± 11.2  MS-LF:  40.0 ± 9.1  HC:  39.2 ± 13.4 | MS-HF: 17/33 MS-LF:  10/19  HC: 9/17 | MS-HF: 2.0 (1.0-4.0)  MS-LF:  1.5 (0.0-4.0) | MS-HF: 12.9 ± 8.2  MS-LF:  10.6 ± 7.6 | MFIS  [>38 (Total)]  MS-HF:  50.4 (38-71)  MS-LF:  22.5 (4-35) | MRI/fMRI  3.0 Tesla  (Intera; Philips Medical Systems) | Normalised brain volume (ml) | 1434 ± 117 | 1448 ± 118 | 1566 ± 296 | Abnormal recruitment of sensorimotor networks in fronto-parietal–temporal lobes and basal ganglia. Normalised brain volume, grey matter volume, white matter volume, T1 and T2 lesion volumes were not significantly different for MS-HF vs MS-LF or HC. |
|  |  |  |  |  |  |  |  |  | Grey matter volume (ml) | 637 ± 80 | 648 ± 80 | 766 ± 58 |  |
|  |  |  |  |  |  |  |  |  | White matter volume (ml) | 795 ± 43 | 800 ± 43 | 858 ± 41 |  |
|  |  |  |  |  |  |  |  |  | T1 Lesion volume (ml) | 4.4 ± 5.6 | 4.1 ± 4.5 |  |  |
|  |  |  |  |  |  |  |  |  | T2 Lesion volume (ml) | 6.2 ± 7.5 | 5.7 ± 6.0 |  |  |
| Roelcke *et al.* (1997) | 37 MS  16 HC | 19 MS-HF  16 MS-LF  16 HC | MS-HF:  43 ± 8  MS-LF:  42 ± 10  HC:  40 ± 15 | MS-HF:  8/11  MS-LF:  7/9  HC: 7/9 | MS-HF: 3.6 ± 1.3  MS-LF: 3.9 ± 1.7 | MS-HF: 10 ± 8  MS-LF:  14 ± 8 | FSS  [>5 (Mean)]  MS-HF:  6.0 ± 0.6  MS-LF:  2.7 ± 0.8 | FDG-PET scans 933/04-16 tomograph (CTI, Knoxville, 4 rings, 7 planes, 8 mm FWHM) | Regional and global cerebral glucose metabolism using PET and 18F-fluorode-oxyglucose. |  |  |  | Reduced cerebral glucose metabolism bilaterally for MS-HF versus MS-LF in the prefrontal area involving the lateral and medial prefrontal cortex and adjacent white matter, in the premotor cortex, putamen, and the right supplementary motor area. Reductions also observed in the white matter extending from the rostral putamen toward the lateral head of the caudate nucleus. Suggests MS fatigue is associated with frontal cortex and basal ganglia dysfunction. |
|  |  |  |  |  |  |  |  |  | Global metabolic rate of glucose (μmol/100 mL/min) | 34.7 ± 4.4 | 35.4 ± 4.5 | 43.3 ± 6.9 |  |
| Sander *et al.* (2016) | 30 RR 12 SP  13 HC | 17 MS-HF  25 MS-LF | MS-HF:  42.8 ± 12.8  MS-LF:  50.5 ± 8.8  HC:  48.6 ± 5 | MS-HF: 10/18,  MS-LF: 5/9  HC: 4/9 | MS-HF: 4.6 ± 1.3  MS-LF:  3.0 ± 2.0 |  | FSMC [≥28 (Cognitive fatigue scale)]  FSMC (Cog):  MS-HF:  36.5 (21)  MS-LF:  21.0 (14)  FSS:  MS-HF:  46 (10.6)  MS-LF:  29 (10.3) | MRI (DTI)  3.0 Tesla scanner (Siemens Skyra) | Brain parenchymal fraction (%) | 81 ± 4 | 81 ± 4 | 85 ± 15 | Brain parenchymal fraction, total brain volume, lateral ventricle volume, third and fourth ventricle volume, T1 lesion volume, axial and radial diffusivity were not significantly different for MS-HF vs MS-LF but differed from HC. |
|  |  |  |  |  |  |  |  |  | T1 lesion volume (ml) | 3.71 ± 2.57 | 7.07 ± 15.77 | 0.03 ± 0.08 |  |
|  |  |  |  |  |  |  |  |  | Total brain volume (ml) | 1201 ± 109 | 1219 ± 131 | 1281 ± 73 |  |
|  |  |  |  |  |  |  |  |  | Lateral ventricles | 40.92 ± 24.68 | 36.00 ± 30.87 | 16.78 ± 6.43 |  |
|  |  |  |  |  |  |  |  |  | Third and fourth ventricle volume | 4.22 ± 1.47 | 3.43 ± 4.46 | 2.72 ± 1.27 |  |
|  |  |  |  |  |  |  |  |  | Corpus callosum index | 0.326 ± 0.067 | 0.329 ± 0.068 | 0.43 ± 0.05 |  |
|  |  |  |  |  |  |  |  |  | Axial and radial diffusivity of the corpus callosum | 0.0013 ± 0.0001 | 0.0013 ± 0.0001 | 0.0012 ± 0.0000 |  |
| Sepulcre *et al.* (2009)^ | 28 RR  5 PP  5 SP  22 CIS  20 HC | 43 MS-HF  17 MS-LF | MS:  36.4 ± 9  HC:  37.4 ± 8.7 | MS: 22/38  HC: 8/12 | MS: 2.0 (0.0-7.0) | MS:  2.7 (1-36) | MFIS-5 (score range = 0-20)  [<5 used to define absence of fatigue]  Means for the 2 MS groups not reported. | MRI  1.5 Tesla scanner (Siemens Symphony) | T1 Lesion volume (ml) | 18.4 ± 6.5 | 6.5 ± 3.2 |  | Greater grey matter atrophy (left superior frontal gyrus, bilateral middle frontal gyrus) in MS-HF vs MS-LF. Higher T1 and T2 lesion volumes for MS-HF vs MS-LF (left frontal and right parieto-temporal white matter regions mainly affected). Reported T1 and T2 gadolinium enhancing lesion volumes, so data were excluded from the meta-analysis. |
|  |  |  |  |  |  |  |  |  | T2 Lesion volume (ml) | 56.6 ± 16.8 | 19.1 ± 8.3 |  |  |
| Specogna *et al.* (2012) | 24 RR  15 HC | 12 MS-HF  12 MS-NF  15 HC | MS-HF: 40.9 ± 8.9  MS-LF:  38.7 ± 8.1 | MS: 4/20 | MS-HF:  Mean=1.5  MS-LF:  Mean=1.5 | MS:  Mean=7 | FSS [>5]  Means for the 2 MS groups not reported. | MRI 1.5 Tesla scanner (Philips Achieva) | Cortical activation during execution of a motor task (sequential finger tapping). | Data presented as brain scans and activation coordinates. | Data presented as brain scans and activation coordinates. | Data presented as brain scans and activation coordinates. | MS-HF demonstrated greater activation of the right premotor area, putamen and dorsolateral prefrontal cortex (i.e. motor attentional network) in comparison with MS-LF. |
| Stefancin *et al.* (2019) | 22 RR | 10 MS-HF  12 MS-LF | MS-HF:  27.0 ± 5.5  MS-LF:  25.8 ± 5.4 | MS-HF:  3/7  MS-LF:  6/4 | MS-HF:  2.3 (0-6)  MS-LF:  1.2 (0-4) | MS-HF:  5.5 ± 3.8  MS-LF:  5.8 ± 3.8 | FSS [>4 (mean)]  MS-HF:  5.5 ± 0.9  MS-LF:  2.0 ± 0.8 | MRI  3T Siemens Biograph mMR | T2 Lesion volume (ml) | 4.7 ± 2.8 | 12.4 ± 19.6 |  | No significant difference in T2 lesion volume between MS-HF and MS-LF. |
| Tartaglia *et al.* (2004) | 60 MS | 34 MS-HF  26 MS-LF | MS-HF:  42.1 ± 6.9  MS-LF:  38.1 ± 10.2 |  | MS-HF: 3.8 ± 2.2  MS-LF: 2.7 ± 2.2 | MS-HF: 10.59 ± 7.3  MS-LF: 10.35 ± 9.5 | FSS  [>5 (Mean)]  RRMS:  4.66 ± 1.5  SPMS:  4.67 ± 1.8 | MRS  1.5 Tesla scanner (Phillips Medical) | T2 Lesion volume (ml) | 13.44 ± 13.4 | 11.24 ± 13.2 |  | Significantly lower NAA/Cr in MS-HF vs MS-LF, suggesting diffuse periventricular axonal injury is associated with MS fatigue. |
|  |  |  |  |  |  |  |  |  | NAA/Cr | 2.69 ± 0.29 | 2.99 ± 0.33 |  |  |
|  |  |  |  |  |  |  |  |  | CHO/Cr | 1.44 ± 0.15 | 1.48 ± 0.18 |  |  |
| Tedeschi *et al.* (2007) | 222 RR | 197 MS-HF  25 MS-LF | MS-HF:  39.0 ± 9.2  MS-LF:  34.0 ± 9.1 |  | EDSS <2 | MS-HF: 10 ± 6.6  MS-LF:  6 ± 5.7 | FSS [≥5 (Mean)]  Means for the 2 groups not reported. | MRI  1.0 Tesla scanner (Genesys Sigma) | Abnormal white matter fraction | 0.02 ± 0.01 | 0.01 ± 0.01 |  | Significantly higher lesion volume, white matter and grey matter atrophy in MS-HF vs MS-LF. |
|  |  |  |  |  |  |  |  |  | White matter fraction | 0.34 ± 0.03 | 0.35 ± 0.03 |  |  |
|  |  |  |  |  |  |  |  |  | Grey matter fraction | 0.49 ± 0.04 | 0.52 ± 0.03 |  |  |
|  |  |  |  |  |  |  |  |  | T1 lesion volume (ml) | 2.6 ± 3.8 | 0.9 ± 1.7 |  |  |
|  |  |  |  |  |  |  |  |  | T2 lesion volume (ml) | 17.5 ± 16.7 | 7.1 ± 6.6 |  |  |
| Tellez *et al.* (2008) | 40 RR  21 HC | 17 MS-HF  13 MS-LF | MS-HF:  38.5 ± 7.6  MS-LF:  37.8 ± 9.8 | MS-HF: 4/13  MS-LF:  3/10 | MS-HF: 2.5 (1.0-3.0)  MS-LF:  1.5 (0.3.0) | MS-HF: 9.3 ± 7.3  MS-LF:  7.0 ± 6.2 | FSS  [≥5 (Mean)]  MS: 4.8 ± 1.5  MS-HF:  5.9 ± 0.7  MS-LF:  3.6 ± 1.15  HC:  3.2 ± 1.2 | MRS  1.5 Tesla scanner (Magnetom Vision) | Frontal white matter lesion volume (%) | 0.12 ± 0.18 | 0.09 ± 0.02 |  | Significantly lower NAA/Cr in the lentiform nucleus region in MS-HF vs MS-LF and HC, indicative of axonal dysfunction. |
|  |  |  |  |  |  |  |  |  | Lentiform nucleus lesion volume (%) | 0.03 ± 0.05 | 0.01 ± 0.04 |  |  |
|  |  |  |  |  |  |  |  |  | NAA/Cr |  |  |  |  |
|  |  |  |  |  |  |  |  |  | Frontal white matter | 1.67 ± 0.17 | 1.73 ± 0.12 | 1.77 ± 0.19 |  |
|  |  |  |  |  |  |  |  |  | Lentiform nucleus | 1.36 ± 0.09 | 1.48 ± 0.10 | 1.47 ± 0.13 |  |
|  |  |  |  |  |  |  |  |  | NAA/Cho |  |  |  |  |
|  |  |  |  |  |  |  |  |  | Frontal white matter | 1.48 ± 0.24 | 1.53 ± 0.17 | 1.45 ± 0.24 |  |
|  |  |  |  |  |  |  |  |  | Lentiform nucleus | 1.86 ± 0.30 | 1.92 ± 0.50 | 1.88 ± 0.40 |  |
|  |  |  |  |  |  |  |  |  | Cho/Cr |  |  |  |  |
|  |  |  |  |  |  |  |  |  | Frontal white matter | 1.14 ± 0.17 | 1.14 ± 0.12 | 1.15 ± 0.33 |  |
|  |  |  |  |  |  |  |  |  | Lentiform nucleus | 0.81 ± 0.13 | 0.76 ± 0.14 | 0.81 ± 0.20 |  |
| Tomasevic *et al.* (2013)** | 20 RR | 11 MS-HF  9 MS-LF | MS-HF:  38.5 ± 3.2  MS-LF:  35.9 ± 7.8 | MS-HF: 3/8  MS-LF: 3/6 | MS-HF: 0.4 ± 0.5  MS-LF: 0.3 ± 0.5 | MS:  4.7 ± 3.8 | MFIS-physical scale  [≥16 (Mean)]  MS-HF:  36.6 ± 10.2  MS-LF:  16.6 ± 8.6 | MRI  1.5 Tesla scanner (Achieva, Phillips) | Brain parenchymal fraction (%) | 82 ± 1 | 81 ± 2 |  | T2 lesion volume, thalamus volume and brain parenchymal fraction were not significantly different for MS-HF vs MS-LF. |
|  |  |  |  |  |  |  |  |  | T2 lesion volume (ml) | 11.0 ± 8.3 | 17.2 ± 8.1 |  |  |
|  |  |  |  |  |  |  |  |  | Lesion relative fraction (%) | 0.02 ± 0.01 | 0.03 ± 0.03 |  |  |
|  |  |  |  |  |  |  |  |  | Thalamus volume (ml): | 14.6 ± 1.8 | 13.4 ± 1.6 |  |  |
|  |  |  |  |  |  |  |  |  | Central sulcus cortical thickness (mm): |  |  |  |  |
|  |  |  |  |  |  |  |  |  | Left | 1.71 ± 0.07 | 1.73 ± 0.18 |  |  |
|  |  |  |  |  |  |  |  |  | Right | 1.70 ± 0.09 | 1.67 ± 0.18 |  |  |
| van der Werf *et al.* (1998) | 26 RR  19 SP | 32 MS-HF  13 MS-LF | MS:  37.6 ± 8.4 | RR: 8/18  SP: 9/10 | MS: Mean=3.5 |  | Feeling tired several times a week; Daily fatigue score (range: 0-16)  Means for the 2 MS groups not reported. | MRI 1.0 Tesla proton density & T2-weighted spin echo | Conventional T1- and T2-weighted MRI provided several measures for cerebral abnormalities. | Data reported as graphs and correlation coefficients. | Data reported as graphs and correlation coefficients. |  | Regional lesion load was not significantly different for MS-HF vs MS-LF. Fatigue severity was not related to the total extent of cerebral abnormalities, or to MRI-based atrophy measures. Suggests factors other than focal lesions or cerebral atrophy mediate levels of perceived MS fatigue. |
| Wilting *et al.* (2016) | 79 MS  40 HC | 38 MS-HF  41 MS-LF  40 HC | MS-HF: 34.5 (20-58)  MS-LF: 30 (17-54) | MS-HF: 8/30  MS-LF:  15/26  HC: 22/18 | MS-HF: 1.5 (0-5.5)  MS-LF: 0.5 (0-3.5) | MS-HF:  2 (0-10)  MS-LF:  2 (0-10) | FSMC [>27 (Cognitive Scale)]  Means for the 2 MS groups not reported. | MRI (DTI) 3 Tesla scanner (Magnetom Tim Trio, Siemens) | Lesion volume (ml) | 3.5 (0.4-41.2) | 1.9 (0.1-30.6) |  | Significant reduction in global grey matter fraction was found for MS-HF versus HC but not MS-LF. Reduced fractional anisotropy and increased mean diffusivity values were found in MS-HF versus MS-LF for the thalamus and basal ganglia, including the caudate nucleus, globus pallidus and putamen. Suggests morphologic and microstructural alterations in thalamic regions are related to cognitive fatigue in early MS. Fractional anisotropy and mean diffusivity values in the thalamus were significantly correlated with information processing speed, cognitive flexibility and overall cognitive impairment. |
|  |  |  |  |  |  |  |  |  | Mean grey matter fraction (%) | 0.440 ± 0.036 | 0.443 ± 0.027 | 0.456 ± 0.025 |  |
|  |  |  |  |  |  |  |  |  | Mean white matter fraction (%) | 0.395 ± 0.025 | 0.392 ± 0.026 | 0.394 ± 0.023 |  |
|  |  |  |  |  |  |  |  |  | Mean cerebrospinal fluid fraction (%) | 0.165 ± 0.028 | 0.164 ± 0.024 | 0.150 ± 0.024 |  |
|  |  |  |  |  |  |  |  |  | Brain parenchymal fraction (%) | 0.835 ± 0.028 | 0.836 ± 0.024 | 0.850 ± 0.024 |  |
|  |  |  |  |  |  |  |  |  | Thalamus fractional anisotropy | 0.275 ± 0.027 | 0.289 ± 0.021 | 0.300 ± 0.015 |  |
|  |  |  |  |  |  |  |  |  | Thalamus mean diffusivity (×10^−3^ mm^2^/s) | 1.426 ± 0.334 | 1.266 ± 0.221 | 1.139± 0.115 |  |
|  |  |  |  |  |  |  |  |  | Basal ganglia fractional anisotropy | 0.252 ± 0.014 | 0.255 ± 0.015 | 0.262 ± 0.012 |  |
|  |  |  |  |  |  |  |  |  | Basal ganglia mean diffusivity (×10^−3^ mm^2^/s) | 0.980 ± 0.146 | 0.912 ± 0.116 | 0.870 ± 0.077 |  |
|  |  |  |  |  |  |  |  |  | Frontal cortex fractional anisotropy | 0.110 ± 0.005 | 0.111 ± 0.006 | 0.115 ± 0.004 |  |
|  |  |  |  |  |  |  |  |  | Thalamus mean diffusivity (×10^−3^ mm^2^/s) | 0.527 ± 0.047 | 0.509 ± 0.039 | 0.490 ± 0.029 |  |
| Yaldizli *et al.* (2011) | 70 RR | 28 MS-HF  42 MS-LF | MS-HF:  43.7 ± 11.4  MS-LF:  40.4 ± 10.5 | MS-HF: 4/24  MS-LF: 5/37 | MS-HF: 3.3 ± 1.2  MS-LF: 2.4 ± 1.7 | MS-HF: 10.7 ± 8.6  MS-LF:  9 ± 6.8 | FSS  [≥4 (Mean)]  MS-HF:  5.27 ± 1.09  MS-LF:  2.1 ± 1.04 | MRI  1.5 Tesla scanner  (Sigma Magnetom) | T2 Lesion load (ml) | 14.0 ± 54.4 | 21.0 ± 55.4 |  | Significantly greater atrophy of corpus callosum in MS-HF vs MS-LF. |
|  |  |  |  |  |  |  |  |  | Black holes on T1weighted MRI: |  |  |  |  |
|  |  |  |  |  |  |  |  |  | Yes | 8 ± 28.6 | 32 ± 76.2 |  |  |
|  |  |  |  |  |  |  |  |  | No | 20 ± 71.4 | 10 ± 23.8 |  |  |
|  |  |  |  |  |  |  |  |  | Contrast enhancing lesions on T1weighted MRI: |  |  |  |  |
|  |  |  |  |  |  |  |  |  | Yes | 1 ± 3.6 | 5 ± 11.9 |  |  |
|  |  |  |  |  |  |  |  |  | No | 27 ± 96.4 | 37 ± 88.1 |  |  |
| Yarraguntla *et al.* (2019) | 30 RR | 16 MS-HF  14 MS-LF | MS-HF:  43 ± 2.9  MS-LF: 39 ± 1.7  HC: | MS-HF:  4/12  MS-LF:  7/7 | MS-HF:  3 ± 0.4  MS-LF:  2.4 ± 0.6 | MS-HF:  10 ± 1.7  MS-LF:  8.6 ± 1.9 | FSS  [≥ 5.1 (mean)]  MS-HF:  6 ± 0.12  MS-LF:  1.89 ± 0.2 | MRI  Siemens 3T Verio MR scanner | T2 lesion volume (ml) | 14 ± 2.5 | 15.3 ± 5.9 |  | No significant difference in T2 lesion volume between MS-HF and MS-LF. |
| Zaini *et al.* (2016) ^ | 19 RR  18 HC | 10 MS-HF  9 MS-LF | MS-HF:  42 ± 8  MS-LF:  38 ± 5  HC:  38 ± 7 | MS-HF: 10/0  MS-LF: 9/0  HC:  18/0 | MS-HF: 1.8 (1.0-2.5)  MS-LF:  1.5 (1.0-1.5) |  | FSS  [>36 (Total)]  MS-HF:  52 ± 6  MS-LF:  22 ± 10  HC:  18 ± 4 | MRI, MRS  1.5 Tesla SCANNER (Siemens Sonata) | T2 lesion volume (ml) | 11.6 ± 14.6 | 5.5 ± 6.4 |  | Lower NAA/Cr concentration in the tegmentum of pons driven by higher Cr concentration in MS-HF vs HC (found in white matter regions). T2 lesion volume data obtained for MS-HF and MS-LF by communication with the lead author. |
| Zellini *et al.* (2009) | 32 RR  13 HC | 23 MS-HF  9 MS-LF | MS-HF:  40 (32-42.5)  MS-LF:  36 (32-39)  HC:  37 (30-43) | MS-HF: 4/19  MS-LF: 1/8  HC:  4/9 | MS-HF:  3 (2.5-4)  MS-LF: 2 (2-2.5) | MS-HF: 10 (3.5-12.5)  MS-LF:  6 (2-12) | FSS  [≥5 (Mean)]  MS-HF:  5.8 (5.25-6.25)  MS-LF:  3.4 (3.22-3.6) | MRI  1.5 Tesla scanner (Vision MR) | T2 lesion volume (ml) | 3.53 (1.66-11.72) | 3.94 (1.98-4.83) |  | T1 and T2 lesion volume was not significantly different for MS-HF vs MS-LF. |

Data are presented as mean ± SD or median with intracortical range or total range in parentheses; ^ Original data received from the lead author; ** article has both structural and function neurophysiological measurements; RR, relapsing-remitting multiple sclerosis; SP, secondary progressive multiple sclerosis; PP, primary progressive multiple sclerosis; RSP, relapsing secondary progressive multiple sclerosis; EDSS, Extended Disability Status Scale; MS, Multiple Sclerosis; MS-HF, multiple sclerosis-highly fatigued; MS-LF, multiple sclerosis-less fatigued; HC, healthy controls; FSS, Fatigue Severity Scale; MFIS, Modified Fatigue Impact Scale; MRI, Magnetic Resonance Imagery, fMRI; Functional Magnetic Resonance Imagery.

**Supplementary Table 3b**. Characteristics of neurophysiological studies included in this review (N=24)

| Author | N (by disease type) | MS Subgroups | Age (y) | Male/ Female | EDSS Scores | Disease  Duration (y) | Perceived Fatigue Measure | Technique | Outcomes | MS-HF | MS-LF | HC | Summary of findings |
| --- | --- | --- | --- | --- | --- | --- | --- | --- | --- | --- | --- | --- | --- |
| Andreasen *et al.* (2009) | 40 RR | 19 MS-HF  21 MS-LF | MS-HF:  43 (27-53)  MS-LF:  39 (23-53) |  | MS-HF:  3.0 (1.0-3.5)  MS-LF:  2.0 (1.5-3.5) | MS-HF:  5.0 (1-14)  MS-LF:  3.0 (0-9) | FSS  [>5 (Mean)]  MS-HF:  6.3 (5.0-7.0)  MS-LF:  3.1 (1.0-4.0) | Biodex System 3 PRO (Biodex Medical Systems);  Stimulator Digitimer model DS7 (Digitimer Ldt) | Maximum voluntary contraction force (N) | 148 ± 32 | 173 ± 49 |  | Significantly lower voluntary activation in MS-HF vs MS-LF. Maximum voluntary contraction force was not significantly different for MS-HF vs MS-LF. |
|  |  |  |  |  |  |  |  |  | Voluntary activation (%) | 95.9 ± 5.1 | 99.2 ± 0.99 |  |  |
|  |  |  |  |  |  |  |  |  | Peripheral activation (%) | 1.02 ± 0.08 | 1.05 ± 0.14 |  |  |
| Andreasen *et al.* (2010)** | 34 RR  7 HC | 17 MS-HF  17 MS-LF | MS-HF:  43 (27-53)  MS-LF:  39 (23-53)  HC:  39 (31-45) | MS-HF: 5/12  MS-LF: 9/8  HC:  1/6 | MS-HF:  3 (1-3.5)  MS-LF:  2 (1.5-3.5)  HC:  0 (0-2) | MS-HF:  5 (1-14)  MS-LF:  3 (0-9) | FSS  [>5 (Mean)]  MS-HF:  6.3 (5-7)  MS-LF:  2.8 (1-4)  HC:  2.7 (2-4) | Isometric dynam-ometer | Voluntary activation (%) | 98.1 (85.1-100) | 99.8 (96.9-100) |  | Significantly lower voluntary activation in MS-HF vs MS-LF. |
| Chalah *et al.* (2019)^ | 6RR  16PP  16SP | 21 MS-HF  17 MS-LF | MS-HF:  51 (44-67)  MS-LF: 53 (34-62) | MS-HF: 9/8  MS-LF: 11/10 | MS-HF:  6.5 (5.5-6.5)  MS-LF:  6.0 (3.0-6.5) | MS-HF:  11.9 ± 6.6  MS-LF:  11.5 ± 5.3 | MFIS [≥45 (Total)]  MS-HF: 58.67 ± 8.85  MS-LF: 32.82 ± 6.61 | TMS  (MC125, Mag Venture) | MEP threshold (%) | 54.7 ± 13.3 | 61.6 ± 18.3 |  | SICI 2 ms (%) was significantly higher in MS-HF versus MS-LF. No difference between MS-HF and MS-LF for SICI 4 ms (%) or ICF 12 ms (%). |
|  |  |  |  |  |  |  |  |  | SICI 2 ms (%) | 63.9 ± 20.9 | 35.0 ± 40.8 |  |  |
|  |  |  |  |  |  |  |  |  | SICI 4 ms (%) | 44.3 ± 37.5 | 9.5 ± 73.3 |  |  |
|  |  |  |  |  |  |  |  |  | ICF 12 ms (%) | 160.8 ± 68.9 | 159.9 ± 78.2 |  |  |
| Cogliati Dezza *et al.* (2015)** | 27 MS  8 HC | 15 MS-HF  12 MS-LF | MS-HF:  37.3 ± 4  MS-LF:  36.9 ± 7.5  HC:  37 (25-48) | MS-HF: 4/11  MS-LF:  4/8  HC:  1/7 | MS-HF:  1 (0-3)  MS-LF:  1 (0-2) | MS-HF:  3.9 ± 4.1  MS-LF:  7.1 ± 3.9 | MFIS  [>36 (Total)]  MS-HF:  42.1 ± 7.3  MS-LF:  19.9 ± 8.6 | EEG  (Micromed S.p.A., Mo- gliano Veneto) | Inter-hemispheric symmetry index at rest: | 1.08 ± 0.08 | 0.99 ± 0.10 | 0.98 ± 0.08 | Significantly higher resting left hemispheric primary sensorimotor activity power and higher inter-hemispheric  coherence during movement in MS-HF vs MS-LF and HC. |
|  |  |  |  |  |  |  |  |  | Left | 2.94 ± 0.70 | 3.28 ± 1.00 | 3.43 ± 1.02 |  |
|  |  |  |  |  |  |  |  |  | Right | 2.53 ± 0.55 | 3.27 ± 0.76 | 3.51 ± 0.86 |  |
|  |  |  |  |  |  |  |  |  | Inter-hemispheric symmetry index - movement: | 1.06 ± 0.34 | 0.99 ± 0.35 | 0.99 ± 0.10 |  |
|  |  |  |  |  |  |  |  |  | Left | 0.27 ± 0.15 | 0.27 ± 0.14 | 0.15 ± 0.08 |  |
|  |  |  |  |  |  |  |  |  | Right | 0.25 ± 0.16 | 0.27 ± 0.11 | 0.16 ± 0.08 |  |
| Colombo *et al.* (2000)** | 30 MS | 15 MS-HF  15 MS-LF | MS-HF:  30.4 (18-49)  MS-LF:  39 (18-49) | MS-HF: 3/12  MS-LF: 4/11 | MS-HF:  1.5 (0-1.5)  MS-LF:  1.5 (0-1.5) | MS-HF:  2.8 (1-7)  MS-LF:  3.7 (1-9) | FSS  [>25 (Total)]  MS-HF:  40 (25-60)  MS-LF:  14 (10-21) | Magstim Stimulator (Cadwell MS10) | Central motor conduction time (ms): |  |  |  | Central motor conduction time was not significantly different for MS-HF vs MS-LF. |
|  |  |  |  |  |  |  |  |  | Right arm | 6.4 (5.3-19.4) | 6.6 (5.5-7.9) |  |  |
|  |  |  |  |  |  |  |  |  | Left arm | 6.7 (5.3-12.3) | 6.8 (5.5-7.7) |  |  |
|  |  |  |  |  |  |  |  |  | Right leg | 16.0 (12.1-31.7) | 15.0 (11.2-16.3) |  |  |
|  |  |  |  |  |  |  |  |  | Left leg | 16.6 (11.1-26.0) | 14.8 (13.0-21.4) |  |  |
| Conte *et al.* (2016) | 25 RR  18 HC | 12 MS-HF  13 MS-LF  18 HC | MS-HF:  41.3 ± 7.7  MS-LF:  38.3 ± 8.4  HC: 37.0 ± 8.0 | MS-HF: 6/6  MS-LF:  6/7  HC: 9/9 | MS-HF: 1.0 (0-3.5)  MS-LF:  1.1 (0-3.5) | MS-HF:  5.2 ± 4.3  MS-LF: 6.3 ± 7.1 | Presence or not of subjective fatigue.  MFIS:  MS-HF:  35.1 ± 10.1  MS-LF: 13.9 ± 8.8 | 5-Hz rTMS  Super Rapid Magstim stimulator (Magstim Co. UK) | 5 Hz rTMS (reflecting short-term plasticity) and paired associative stimulation (reflecting long-term plasticity) during 2 different attention-demanding conditions. | Data reported as graphs. | Data reported as graphs. | Data reported as graphs. | Effects of attention on cortical plasticity differ in MS-HF versus MS-LF. In MS-LF attention improves the MEP size increase whereas in patients with fatigue, attention leaves responses unchanged.  Suggests MS fatigue reflects disrupted cortical attentional networks related to movement control. |
| Greim *et al.* (2007) | 76 RR  51 HC | 46 MS-HF  30 MS-LF | MS:  36.7 ± 8.6  HC:  35.2 ± 12.6 | MS: 14/65  HC: 15/35 | MS:  2.51 ± 1.89 | MS:  6.9 ± 4.3 | MS-HF: Mostly or daily tired  MS-LF: Rarely or occasion-ally tired | Hand-dynam-ometer | Post fatigue task force (% baseline force) | 87.5 ± 11.6 | 95.6 ± 12.9 | 96.8 ± 8.4 | Greater fatigability in MS-HF vs MS-LF and HC. |
| Leocani *et al.* (2001) | 33 RR  14 HC | 15 MS-HF  18 MS-LF  14 HC | MS-HF: 33 ± 8  MS-LF:  32 ± 6 | MS-HF: 3/12  MS-LF:  5/13 | ≤1.5 |  | FSS [≥33 (Total)]  Means for the 2 MS groups not reported. | 29 channel EEG | Event-related desynch-ronisation (ERD)/event-related synch-ronisation (ERS) of the 10 and 18-22 Hz bands (cortical circuits involved in control of voluntary movement). | Data reported as average topographic maps and regression lines. | Data reported as average topographic maps and regression lines. | Data reported as average topographic maps and regression lines. | Reduced post-movement 18 –22 Hz ERS in MS-HF versus MS-LF and inverse correlation between the amount of ERS and the fatigue score. Suggests inhibitory circuits acting on the motor cortex after movement termination may be involved in the patho-physiological mechanism of MS fatigue. |
| Liepert *et al.* (2005) | 16 RR  6 HC | 8 MS-HF  8 MS-LF | MS-HF:  42.5 ± 5  MS-LF:  40.3 ± 4.5  HC:  32.8 ± 10.3 | MS-HF: 1/7  MS-LF: 2/6  HC:  6/0 | MS-HF:  3.1 ± 0.93  MS-LF:  2.9 ± 0.9 |  | FSS [≥ 4 (Mean)]  MS-HF:  5.3 ± 0.4  MS-LF:  1.1 ± 0.2 | Bistim device (Magstim Comp) | Resting motor threshold (%) | 46.0 ± 7 | 46.2 ± 3.6 | 40.9 ± 6.1 | Significant reductions in SICI 2-3 ms (%) at rest in MS-HF vs MS-LF and HC. Significantly lower grip strength in MS-HF vs HC. Motor response, resting motor threshold, motor evoked potential amplitude and latency was not different for MS-HF vs MS-LF or HC. |
|  |  |  |  |  |  |  |  |  | Motor evoked potential amplitude (mV) | 0.54 ± 0.26 | 0.57 ± 0.19 | 0.65 ± 0.27 |  |
|  |  |  |  |  |  |  |  |  | Motor evoked potential latency (ms) | 15.7 ± 1.23 | 15.7 ± 1.56 | 15.3 ± 1.24 |  |
|  |  |  |  |  |  |  |  |  | SICI 2-3 ms (%) | 54.6 ± 27.3 | 35.9 ± 10.2 | 31.2 ± 14 |  |
|  |  |  |  |  |  |  |  |  | ICF 11-13 ms (%) | 168 ± 37 | 179 ± 39 | 150 ± 35 |  |
|  |  |  |  |  |  |  |  |  | Motor response (mV) | 18.6 ± 3.8 | 14.4 ± 6.5 | 19.5 ± 4.8 |  |
|  |  |  |  |  |  |  |  |  | Grip strength (Nm) | 87 ± 20.2 | 102.5 ± 21.1 | 120 ± 14.4 |  |
| Morgante *et al.* (2011)** | 33 RR  12 HC | 16 MS-HF  17 MS-LF | MS-HF:  41.1 ± 10.9  MS-LF:  38 ± 9.4 | MS-HF: 7/9  MS-LF: 4/13 | MS-HF:  1.8 ± 0.6  MS-LF:  1.6 ± 0.6 | MS-HF:  8.4 ± 3.4  MS-LF:  7.9 ± 3.8 | FSS [>4 (Mean)]  MS-HF:  4.9 ± 0.8  MS-LF:  2.2 ± 0.9 | Magstim 200 Stimulator and Bistem module  (Magstim Company Ltd)  Neurolog system (Digitimer Ldt) | Central motor conduction time (ms) | 7.3 ± 4.0 | 7.7 ± 6.2 | 5.6 ± 3.42 | Significantly reduced pre-movement facilitation in MS-HF vs MS-LF and HC. Central motor conduction time was prolonged in both MS groups vs HC. SICI 2 ms (%) and ICF 10 ms (%) was not significantly different for MS-HF vs MS-LF or HC. |
|  |  |  |  |  |  |  |  |  | SICI 2 ms (%) | 54 ± 40 | 47 ± 29 | 54 ± 21 |  |
|  |  |  |  |  |  |  |  |  | ICF 10 ms (%) | 132 ± 40 | 123 ± 41 | 140 ± 35 |  |
| Ng *et al.* (2000)^ | 9 MS  11 HC | 6 MS-HF  3 MS-LF | MS: 46 ± 1  HC: 43 ± 2 | MS:  4/5  HC:  6/5 | MS:  2 (1.5-4.5) |  | FSS [≥4 (Mean)]  MS-HF:  5.6 ± 1.5  MS-LF:  3.0 ± 0.6  HC:  3.0 ± 0.4 | Tailor-made force trans-ducer | Maximum voluntary contraction (N) | 182.8 ± 60.7 | 166.9 ± 7.9 | 292 ± 123 | Small group of PwMS and MS-LF (N=3), not justifying statistical comparisons. Data were acquired from the senior author. |
|  |  |  |  |  |  |  |  |  | Voluntary activation (%) | 97.2 ± 4.9 | 98.7 ± 2.3 | 100 (96-100) |  |
|  |  |  |  |  |  |  |  |  | Post fatigue task force (% baseline force) | 83.0 ± 25.3 | 49.8 ± 12.3 | 56 ± 20 |  |
| Ng *et al*. (2004)^ | 16 MS  18 HC | 11 MS-HF  5 MS-LF | MS: 44 ± 2  HC: 47 ± 1 | MS: 5/11  HC: 6/12 | MS-HF:  3.4 ± 1.7  MS-LF:  2.7 ± 1.6 |  | FSS [>4 (Mean)]  MS-HF:  5.8 ± 0.8  MS-LF:  3.1 ± 0.8  HC  2.9 ± 0.2 | Tailor-made force trans-ducer NS6 stimulator (Teca) | Maximum voluntary contraction force (N) | 116.8 ± 62.9 | 126.3 ± 53.6 | 157 ± 51 | Lower maximum voluntary contraction force and muscle activation in MS-HF vs MS-LF and HC. |
|  |  |  |  |  |  |  |  |  | Muscle activation (%) | 85.1 ± 23.8 | 89.2 ± 22.5 | 96.0 ± 13.0 |  |
| Perretti *et al.* (2004) | 41 RR  13 HC | 32 MS-HF  9 MS-LF | MS-HF:  37.7 ± 10  MS-LF:  28.7 ± 7.1  HC:  30.7 ± 8.8 | MS-HF: 18/14  MS-LF: 2/7  HC:  5/8 | MS-HF:  3.4 ± 1.0  MS-LF:  2.3 ± 0.5 |  | FSS [>37 (Total)]  MS-HF:  51.6 ± 8.5  MS-LF:  25.1 ± 11.8  HC:  24.9 ± 6.4 | Dynam-ometer (Pinch Gauge, B and L Engin-eering)  MagPro Dantec Stimulator | Maximum voluntary contraction force (N) | 85.3 ± 14.7 | 93.2 ± 25.5 | 90.2 ± 20.6 | Maximum voluntary contraction force, motor evoked potential amplitude, threshold, latency, duration, and post-exercise MEP facilitation were not significantly different between MS-HF and MS-LF. |
|  |  |  |  |  |  |  |  |  | Motor evoked potential threshold (%) | 72.5 ± 9.1 | 67.8 ± 10.9 | 61.5 ± 8.0 |  |
|  |  |  |  |  |  |  |  |  | Motor evoked potential amplitude (mV) | 0.79 ± 0.89 | 1.32 ± 1.25 | 2.33 ± 1.75 |  |
|  |  |  |  |  |  |  |  |  | Motor evoked potential latency (ms) | 28.9 ± 5.6 | 25.0 ± 3.6 | 23.5 ± 2.1 |  |
|  |  |  |  |  |  |  |  |  | Motor evoked potential duration (ms) | 21.4 ± 6.8 | 16.9 ± 4.3 | 15.9 ± 3.9 |  |
| Romani *et al.* (2004) | 60 MS | 40 MS-HF  20 MS-LF | MS:  38.3 ± 8.1 | MS-HF: 18/22  MS-LF:  9/11 | MS-HF: 2.6 ± 1.4  MS-LF:  2.7 ± 1.7 | MS-HF:  4.5 ± 2.4  MS-LF:  4.7 ± 2.3 | FSS  MS-HF>5.6  MS-LF<2.4 | Force transducer, which measured thumb isometric adduction force. | Fatigability expressed as decline in force and voluntary activation after 45 s sustained MVC. | Data presented as scatterplots and correlation coefficients. | Data presented as scatterplots and correlation coefficients. |  | FSS fatigue scores did not correlate with fatigability. Suggests perceived MS fatigue is independent of fatigability. |
| Russo *et al.* (2015) | 24 RR  10 HC | 12 MS-HF  12 MS-LF  10 HC | MS-HF:  41 ± 7  MS-LF:  39 ± 9 | MS-HF: 5/7  MS-LF: 7/5 | MS-HF:  2.0 ± 1.0  MS-LF:  2.0 ± 1.0 |  | FSS [≥36 (Total)]  MS-HF:  50 ± 7  MS-LF:  20 ± 11 | TMS  (Magstim 200 Co) | Motor cortex excitability and the pre-movement facilitation (PMF) through TMS before and after 5 min of sequenced finger-tapping movements at a fixed frequency of 2 Hz. | Data presented as graphs. | Data presented as graphs. | Data presented as graphs. | Post-task PMF was significantly decreased in MS-HF versus MS-LF and abnormalities were correlated with the performance decay. Suggests possible link between MS fatigue and functional impairment within circuits engaged in movement preparation, upstream the corticospinal tract. |
| Scheidegger *et al.* (2012)^ | 23 MS  13 HC | 10 MS-HF  13 MS-LF | MS:  39.7 ± 11.4  HC:  28 (23-54) | MS: 19/4  HC: 10/3 | MS:  3.15 ± 1.56 |  | FSS [>36 (Total)]  MS:  37.2 ± 14.6 | Force transducer (Sensotec Inc) | Post fatigue task force (% baseline force) | 35.2 ± 18.6 | 36.6 ± 15.0 | 44 ± 9 | Fatigability was not significantly different between MS-HF vs MS-LF. |
| Sebastiao *et al.* (2017) | 62 RR | 36 MS-HF  26 MS-LF | MS-HF:  52.7 ± 6.9  MS-LF:  51.3 ± 8.8 | MS-HF: 9/27  MS-LF: 8/18 | MS-HF:  4.5 (2.0)  MS-LF:  3.5 (3.5) | MS-HF:  13.9 ± 9.1  MS-LF:  12.2 ± 8.3 | MFIS [>38 (Total)]  MS:  4.5 (2.5)  MS-HF:  3.5 (3.5)  MS-LF:  4.5 (2.0) | Isometric Dynam-ometer  (Biodex System 3) | Bilateral isometric peak torque (Nm) |  |  |  | Significantly lower knee flexor peak torque and cardiorespiratory capacity in MS-HF vs MS-LF. |
|  |  |  |  |  |  |  |  |  | Knee flexor | 51.5 ± 19.9 | 66.9 ± 28.6 |  |  |
|  |  |  |  |  |  |  |  |  | Knee extensor | 139.8 ± 47.3 | 165.1 ± 58.3 |  |  |
| Severijns *et al.*  (2019) ^ | 13 RR  5 SP  1 PP | 13 MS-HF  6 MS-LF | MS: 52 ± 9.3  HC: 52 ± 9.2 | MS: 7/12  HC: 7/12 | MS:  3.0 (1.5-6.5) | MS:  15.6 ± 9.2 | FSS [>4 (Mean)]  MS:  4.55 ± 1.60  HC:  2.29 ± 0.80 | Tailor-made force sensor attached to bar | Voluntary activation (%) | 92.7 ± 6.7 | 97.2 ± 1.7 |  | Lower voluntary activation in MS-HF vs MS-LF. |
| Steens *et al.* (2012)^ | 20 MS  20 HC | 18 MS-HF  2 MS-LF | MS: 20-58  HC: 21-57 | MS: 7/13  HC: 6/14 | MS-HF:  2.8 (0-5.0)  MS-LF:  1.0 (0-2.0) | MS:  4 (1-23) | FSS [>4 (Mean)]  MS-HF:  5.5 ± 0.8  MS-LF:  3.7 ± 0.2  HC:  2.9 ± 0.6 | Force transducer  Stimulator Digitimer model DS7 (Digitimer Ltd);  Magstim 200 Stimulator | Maximum voluntary contraction force (N) | 30.7 ± 9.9 | 30.5 ± 6.4 | 34.8 ± 9.3 | Lower voluntary activation in MS-HF vs MS-LF and HC. Maximum voluntary contraction force and fatigability was not significantly different between MS-HF vs MS-LF and HC. |
|  |  |  |  |  |  |  |  |  | Post fatigue task force (% baseline force) | 36.3 ± 13.1 | 33.8 ± 5.0 | 36.6 ± 11.9 |  |
|  |  |  |  |  |  |  |  |  | Voluntary activation (%) | 92.1 ± 7.9 | 99.0 ± 1.5 | 96.7 (81.9-99.1) |  |
|  |  |  |  |  |  |  |  |  | Central motor conduction time (ms) | 9.8 ± 2.6 | 10.7 ± 1.5 | 8.2 (6.2-9.8) |  |
| Tomasevic *et al.* (2013)** | 20 RR | 11 MS-HF  9 MS-LF | MS-HF:  38.5 ± 3.2  MS-LF:  35.9 ± 7.8 | MS-HF: 3/8  MS-LF: 3/6 | MS-HF:  0.4 ± 0.5  MS-LF:  0.3 ± 0.5 | MS:  4.7 ± 3.8 | MFIS-physical scale  [≥16 (Mean)]  MS-HF:  36.6 ± 10.2  MS-LF:  16.6 ± 8.6 | EEG  (Micromed System Plus SAM32 (Micromed) | Cortico-muscular coherence (CMC) | 27.5 ± 4.8 | 16.7 ± 3.6 |  | Significantly faster frequencies of cortico-muscular coherence and increase correction rate during handgrip in MS-HF vs MS-LF. |
|  |  |  |  |  |  |  |  |  | Frequency | 0.07 ± 0.02 | 0.06 ± 0.05 |  |  |
|  |  |  |  |  |  |  |  |  | Amplitude | 0.03 ± 0.02 | 0.05 ± 0.04 |  |  |
|  |  |  |  |  |  |  |  |  | Task performance  correction rate | 1.40 ± 0.38 | 2.20 ± 0.55 |  |  |
| Vecchio *et al.* (2017) | 27 RR  11 HC | 16 MS-HF  11 MS-LF | MS-HF:  37.3 ± 4.0  MS-LF:  36.9 ± 7.5  HC:  36 (28-49) | MS-HF: 3/13  MS-LF: 4/7  HC:  2/9 | MS-HF:  0.5 (0-2)  MS-LF:  1 (0-2) | MS-HF:  4.9 ± 4.1  MS-LF:  5.7± 3.9 | MFIS [>35 (Total)]  MS-HF:  40.8 ± 13.0  MS-LF:  23.4 ± 6.2 | EEG Model CUEE60M, Sei EMG srl Cittadella Italy | Alpha 2 band (10.5-13 Hz) | 1.014 ± 0.00 | 0.995 ± 0.011 | 0.989 ± 0.005 | Functional connectivity changes of the left sensory cortical network at rest, mediated by beta band oscillatory activity in MS-HF vs MS-LF. |
|  |  |  |  |  |  |  |  |  | Beta 1 band (13-20 Hz) | 1.007 ± 0.004 | 0.989 ± 0.009 | 0.985 ± 0.005 |  |
| Wolkorte *et al.* (2015a)^ | 82 RR | 61 MS-HF  21 MS-LF | MS-HF:  41 (21-65)  MS-LF:  42 (25-64) | MS: 32/51 |  | MS:  9.3 (0-34) | FSS [>4 (Mean)]  MS-HF:  5.3 ± 0.6  MS-LF:  2.6 ± 0.8 | Tailor-made force trans-ducer | Maximum voluntary contraction force (N) | 26.33 ± 9.17 | 30.78 ± 9.57 |  | Lower maximum voluntary contraction force and greater fatigability in MS-HF vs MS-LF. |
|  |  |  |  |  |  |  |  |  | Post fatigue task force (% baseline force) | 27.47 ± 8.52 | 36.03 ± 12.40 |  |  |
| Wolkorte *et al.* (2015b)^ | 16 RR  18 HC | 8 MS-HF  8 MS-LF | MS:  39 (21-57)  HC:  38 (21-54) | MS: 11/7  HC: 11/7 | MS:  1.2 (0-3.0)  MS-HF:  1.4 ± 1.0  MS-LF:  0.7 ± 0.9 | MS:  5.5 (1-16) | FSS [>4 (Mean)]  MS:  3.9 (1.6-6.2)  HC:  2.4 (1.3-4.6) | Tailor-made force trans-ducer | Maximum voluntary contraction force (N) | 41.62 ± 24.62 | 37.87 ± 8.16 | 45 ± 11 | Maximum voluntary contraction force was not significantly different between MS-HF vs MS-LF or HC. |
| Wolkorte *et al.* (2016)^ | 25 SP  25 HC | 21 MS-HF  4 MS-LF | SPMS:  53 (41-65)  HC:  53 (40-63) | SPMS: 8/17  HC: 8/17 | MS-HF:  4.9 ± 1.4  MS-LF:  5.6 ± 1.8 | SPMS:  15 (4-37) | FSS [>4 (Mean)]  MS-HF:  5.6 ± 0.7  MS-LF:  2.6 ± 0.6  HC:  2.5 (1.3-4.3) | Tailor-made force trans-ducer  Digitimer model DS7 (Digitimer Ltd) | Maximum voluntary contraction (N) | 24.3 ± 10.3 | 27.4 ± 7.0 | 32.1 ± 9.6 | Maximum voluntary contraction force was similar between MS-HF and MS-LF. Greater fatigability in MS-HF vs MS-LF. |
|  |  |  |  |  |  |  |  |  | Voluntary activation (%) | 83.7 ± 12.3 | 89.3 ± 9.7 | 93.9 ± 5.8 |  |
|  |  |  |  |  |  |  |  |  | Post fatigue task force (% baseline force) | 26.5 ± 8.5 | 30.9 ± 13.9 | 37.2 ± 12.3 |  |

Data are presented as mean ± SD or median with intracortical range or total range in parentheses; ^ Original data received from the lead author; ** article has both structural and function neurophysiological measurements; RR, relapsing-remitting multiple sclerosis; SP, secondary progressive multiple sclerosis; PP, primary progressive multiple sclerosis; RSP, relapsing secondary progressive multiple sclerosis; EDSS, Extended Disability Status Scale; MS, Multiple Sclerosis; MS-HF, multiple sclerosis-highly fatigued; MS-LF, multiple sclerosis-less fatigued; HC, healthy controls; FSS, Fatigue Severity Scale; MFIS, Modified Fatigue Impact Scale; MRI, Magnetic Resonance Imagery, fMRI; Functional Magnetic Resonance Imagery; SICI, short-interval intracortical inhibition; ICF, intracortical facilitation; MEP, motor-evoked potential.

**Reference list for included studies**

Andreasen, A.K., Jakobsen, J., Petersen, T., Andersen, H., 2009. Fatigued patients with multiple sclerosis have impaired central muscle activation. Mult Scler 15, 818-827.

Andreasen, A.K., Jakobsen, J., Soerensen, L., Andersen, H., Petersen, T., Bjarkam, C.R., Ahdidan, J., 2010. Regional brain atrophy in primary fatigued patients with multiple sclerosis. Neuroimage 50, 608-615.

Bakshi, R., Miletich, R.S., Henschel, K., Shaikh, Z.A., Janardhan, V., Wasay, M., Stengel, L.M., Ekes, R., Kinkel, P.R., 1999. Fatigue in multiple sclerosis: cross-sectional correlation with brain MRI findings in 71 patients. Neurology 53, 1151-1153.

Bernitsas, E., Yarraguntla, K., Bao, F., Sood, R., Santiago-Martinez, C., Govindan, R., Khan, O., Seraji-Bozorgzad, N., 2017. Structural and Neuronal Integrity Measures of Fatigue Severity in Multiple Sclerosis. Brain Sci 7.

Bisecco, A., Caiazzo, G., d'Ambrosio, A., Sacco, R., Bonavita, S., Docimo, R., Cirillo, M., Pagani, E., Filippi, M., Esposito, F., Tedeschi, G., Gallo, A., 2016. Fatigue in multiple sclerosis: The contribution of occult white matter damage. Mult Scler 22, 1676-1684.

Bisecco, A., Nardo, F.D., Docimo, R., Caiazzo, G., d'Ambrosio, A., Bonavita, S., Capuano, R., Sinisi, L., Cirillo, M., Esposito, F., Tedeschi, G., Gallo, A., 2017. Fatigue in multiple sclerosis: The contribution of resting-state functional connectivity reorganization. Mult Scler, 1352458517730932.

Calabrese, M., Rinaldi, F., Grossi, P., Mattisi, I., Bernardi, V., Favaretto, A., Perini, P., Gallo, P., 2010. Basal ganglia and frontal/parietal cortical atrophy is associated with fatigue in relapsing-remitting multiple sclerosis. Mult Scler 16, 1220-1228.

Chalah, M.A., Kauv, P., Creange, A., Hodel, J., Lefaucheur, J.P., Ayache, S.S., 2019. Neurophysiological, radiological and neuropsychological evaluation of fatigue in multiple sclerosis. Mult Scler Relat Disord 28, 145-152.

Codella, M., Rocca, M.A., Colombo, B., Rossi, P., Comi, G., Filippi, M., 2002. A preliminary study of magnetization transfer and diffusion tensor MRI of multiple sclerosis patients with fatigue. J Neurol 249, 535-537.

Cogliati Dezza, I., Zito, G., Tomasevic, L., Filippi, M.M., Ghazaryan, A., Porcaro, C., Squitti, R., Ventriglia, M., Lupoi, D., Tecchio, F., 2015. Functional and structural balances of homologous sensorimotor regions in multiple sclerosis fatigue. J Neurol 262, 614-622.

Colombo, B., Martinelli Boneschi, F., Rossi, P., Rovaris, M., Maderna, L., Filippi, M., Comi, G., 2000. MRI and motor evoked potential findings in nondisabled multiple sclerosis patients with and without symptoms of fatigue. J Neurol 247, 506-509.

Conte, A., Li Voti, P., Pontecorvo, S., Quartuccio, M.E., Baione, V., Rocchi, L., Cortese, A., Bologna, M., Francia, A., Berardelli, A., 2016. Attention-related changes in short-term cortical plasticity help to explain fatigue in multiple sclerosis. Mult Scler 22, 1359-1366.

Cruz Gomez, A.J., Ventura Campos, N., Belenguer, A., Avila, C., Forn, C., 2013. Regional brain atrophy and functional connectivity changes related to fatigue in multiple sclerosis. PLoS One 8, e77914.

Damasceno, A., Damasceno, B.P., Cendes, F., 2016. Atrophy of reward-related striatal structures in fatigued MS patients is independent of physical disability. Mult Scler 22, 822-829.

Derache, N., Grassiot, B., Mezenge, F., Emmanuelle Dugue, A., Desgranges, B., Constans, J.M., Defer, G.L., 2013. Fatigue is associated with metabolic and density alterations of cortical and deep gray matter in Relapsing-Remitting-Multiple Sclerosis patients at the earlier stage of the disease: A PET/MR study. Mult Scler Relat Disord 2, 362-369.

Dobryakova, E., Hulst, H.E., Spirou, A., Chiaravalloti, N.D., Genova, H.M., Wylie, G.R., DeLuca, J., 2018. Fronto-striatal network activation leads to less fatigue in multiple sclerosis. Mult Scler 24, 1174-1182.

Filippi, M., Rocca, M.A., Colombo, B., Falini, A., Codella, M., Scotti, G., Comi, G., 2002. Functional magnetic resonance imaging correlates of fatigue in multiple sclerosis. Neuroimage 15, 559-567.

Gobbi, C., Rocca, M.A., Pagani, E., Riccitelli, G.C., Pravata, E., Radaelli, M., Martinelli-Boneschi, F., Falini, A., Copetti, M., Comi, G., Filippi, M., 2014a. Forceps minor damage and co-occurrence of depression and fatigue in multiple sclerosis. Mult Scler 20, 1633-1640.

Gobbi, C., Rocca, M.A., Riccitelli, G., Pagani, E., Messina, R., Preziosa, P., Colombo, B., Rodegher, M., Falini, A., Comi, G., Filippi, M., 2014b. Influence of the topography of brain damage on depression and fatigue in patients with multiple sclerosis. Mult Scler 20, 192-201.

Gonzalez Campo, C., Salamone, P.C., Rodriguez-Arriagada, N., Richter, F., Herrera, E., Bruno, D., Pagani Cassara, F., Sinay, V., Garcia, A.M., Ibanez, A., Sedeno, L., 2019. Fatigue in multiple sclerosis is associated with multimodal interoceptive abnormalities. Mult Scler, 1352458519888881.

Greim, B., Benecke, R., Zettl, U.K., 2007. Qualitative and quantitative assessment of fatigue in multiple sclerosis (MS). J Neurol 254 Suppl 2, II58-64.

Hanken, K., Eling, P., Kastrup, A., Klein, J., Hildebrandt, H., 2015. Integrity of hypothalamic fibers and cognitive fatigue in multiple sclerosis. Mult Scler Relat Disord 4, 39-46.

Hanken, K., Eling, P., Klein, J., Klaene, E., Hildebrandt, H., 2016. Different cortical underpinnings for fatigue and depression in MS? Mult Scler Relat Disord 6, 81-86.

Hidalgo de la Cruz, M., d'Ambrosio, A., Valsasina, P., Pagani, E., Colombo, B., Rodegher, M., Falini, A., Comi, G., Filippi, M., Rocca, M.A., 2018. Abnormal functional connectivity of thalamic sub-regions contributes to fatigue in multiple sclerosis. Mult Scler 24, 1183-1195.

Jaeger, S., Paul, F., Scheel, M., Brandt, A., Heine, J., Pach, D., Witt, C.M., Bellmann-Strobl, J., Finke, C., 2018. Multiple sclerosis-related fatigue: Altered resting-state functional connectivity of the ventral striatum and dorsolateral prefrontal cortex. Mult Scler, 1352458518758911.

Leocani, L., Colombo, B., Magnani, G., Martinelli-Boneschi, F., Cursi, M., Rossi, P., Martinelli, V., Comi, G., 2001. Fatigue in multiple sclerosis is associated with abnormal cortical activation to voluntary movement--EEG evidence. Neuroimage 13, 1186-1192.

Liepert, J., Mingers, D., Heesen, C., Baumer, T., Weiller, C., 2005. Motor cortex excitability and fatigue in multiple sclerosis: a transcranial magnetic stimulation study. Mult Scler 11, 316-321.

Lin, F., Zivadinov, R., Hagemeier, J., Weinstock-Guttman, B., Vaughn, C., Gandhi, S., Jakimovski, D., Hulst, H.E., Benedict, R.H., Bergsland, N., Fuchs, T., Dwyer, M.G., 2019. Altered nuclei-specific thalamic functional connectivity patterns in multiple sclerosis and their associations with fatigue and cognition. Mult Scler 25, 1243-1254.

Morgante, F., Dattola, V., Crupi, D., Russo, M., Rizzo, V., Ghilardi, M.F., Terranova, C., Girlanda, P., Quartarone, A., 2011. Is central fatigue in multiple sclerosis a disorder of movement preparation? J Neurol 258, 263-272.

Ng, A.V., Dao, H.T., Miller, R.G., Gelinas, D.F., Kent-Braun, J.A., 2000. Blunted Pressor and Intramuscular Metabolic Responses to Voluntary Isometric Exercise in Multiple Sclerosis. Cardiopulmonary Physical Therapy Journal 11, 110-111.

Ng, A.V., Miller, R.G., Gelinas, D., Kent-Braun, J.A., 2004. Functional relationships of central and peripheral muscle alterations in multiple sclerosis. Muscle Nerve 29, 843-852.

Niepel, G., Tench Ch, R., Morgan, P.S., Evangelou, N., Auer, D.P., Constantinescu, C.S., 2006. Deep gray matter and fatigue in MS: a T1 relaxation time study. J Neurol 253, 896-902.

Pardini, M., Bonzano, L., Mancardi, G.L., Roccatagliata, L., 2010. Frontal networks play a role in fatigue perception in multiple sclerosis. Behav Neurosci 124, 329-336.

Pellicano, C., Gallo, A., Li, X., Ikonomidou, V.N., Evangelou, I.E., Ohayon, J.M., Stern, S.K., Ehrmantraut, M., Cantor, F., McFarland, H.F., Bagnato, F., 2010. Relationship of cortical atrophy to fatigue in patients with multiple sclerosis. Arch Neurol 67, 447-453.

Perretti, A., Balbi, P., Orefice, G., Trojano, L., Marcantonio, L., Brescia-Morra, V., Ascione, S., Manganelli, F., Conte, G., Santoro, L., 2004. Post-exercise facilitation and depression of motor evoked potentials to transcranial magnetic stimulation: a study in multiple sclerosis. Clin Neurophysiol 115, 2128-2133.

Pravata, E., Zecca, C., Sestieri, C., Caulo, M., Riccitelli, G.C., Rocca, M.A., Filippi, M., Cianfoni, A., Gobbi, C., 2016. Hyperconnectivity of the dorsolateral prefrontal cortex following mental effort in multiple sclerosis patients with cognitive fatigue. Mult Scler 22, 1665-1675.

Riccitelli, G., Rocca, M.A., Forn, C., Colombo, B., Comi, G., Filippi, M., 2011. Voxelwise Assessment of the Regional Distribution of Damage in the Brains of Patients with Multiple Sclerosis and Fatigue. American Journal of Neuroradiology 32, 874-879.

Rocca, M.A., Absinta, M., Valsasina, P., Copetti, M., Caputo, D., Comi, G., Filippi, M., 2012. Abnormal cervical cord function contributes to fatigue in multiple sclerosis. Multiple Sclerosis Journal 18, 1552-1559.

Rocca, M.A., Gatti, R., Agosta, F., Broglia, P., Rossi, P., Riboldi, E., Corti, M., Comi, G., Filippi, M., 2009. Influence of task complexity during coordinated hand and foot movements in MS patients with and without fatigue. A kinematic and functional MRI study. J Neurol 256, 470-482.

Rocca, M.A., Meani, A., Riccitelli, G.C., Colombo, B., Rodegher, M., Falini, A., Comi, G., Filippi, M., 2016. Abnormal adaptation over time of motor network recruitment in multiple sclerosis patients with fatigue. Mult Scler 22, 1144-1153.

Rocca, M.A., Parisi, L., Pagani, E., Copetti, M., Rodegher, M., Colombo, B., Comi, G., Falini, A., Filippi, M., 2014. Regional but not global brain damage contributes to fatigue in multiple sclerosis. Radiology 273, 511-520.

Roelcke, U., Kappos, L., Lechner-Scott, J., Brunnschweiler, H., Huber, S., Ammann, W., Plohmann, A., Dellas, S., Maguire, R.P., Missimer, J., Radu, E.W., Steck, A., Leenders, K.L., 1997. Reduced glucose metabolism in the frontal cortex and basal ganglia of multiple sclerosis patients with fatigue: a 18F-fluorodeoxyglucose positron emission tomography study. Neurology 48, 1566-1571.

Romani, A., Bergamaschi, R., Candeloro, E., Alfonsi, E., Callieco, R., Cosi, V., 2004. Fatigue in multiple sclerosis: multidimensional assessment and response to symptomatic treatment. Mult Scler 10, 462-468.

Russo, M., Crupi, D., Naro, A., Avanzino, L., Buccafusca, M., Dattola, V., Terranova, C., Sottile, F., Rizzo, V., Ghilardi, M.F., Girlanda, P., Bove, M., Quartarone, A., 2015. Fatigue in patients with multiple sclerosis: from movement preparation to motor execution. J Neurol Sci 351, 52-57.

Sander, C., Eling, P., Hanken, K., Klein, J., Kastrup, A., Hildebrandt, H., 2016. The Impact of MS-Related Cognitive Fatigue on Future Brain Parenchymal Loss and Relapse: A 17-Month Follow-up Study. Frontiers in Neurology 7.

Scheidegger, O., Kamm, C.P., Humpert, S.J., Rosler, K.M., 2012. Corticospinal output during muscular fatigue differs in multiple sclerosis patients compared to healthy controls. Mult Scler 18, 1500-1506.

Sebastiao, E., Hubbard, E.A., Klaren, R.E., Pilutti, L.A., Motl, R.W., 2017. Fitness and its association with fatigue in persons with multiple sclerosis. Scand J Med Sci Sports 27, 1776-1784.

Sepulcre, J., Masdeu, J.C., Goni, J., Arrondo, G., Velez de Mendizabal, N., Bejarano, B., Villoslada, P., 2009. Fatigue in multiple sclerosis is associated with the disruption of frontal and parietal pathways. Mult Scler 15, 337-344.

Severijns, D., Cuypers, K., Meesen, R., Feys, P., Zijdewind, I., 2019. Force decline after low and high intensity contractions in persons with multiple sclerosis. Clin Neurophysiol 130, 359-367.

Specogna, I., Casagrande, F., Lorusso, A., Catalan, M., Gorian, A., Zugna, L., Longo, R., Zorzon, M., Naccarato, M., Pizzolato, G., Ukmar, M., Cova, M.A., 2012. Functional MRI during the execution of a motor task in patients with multiple sclerosis and fatigue. Radiol Med 117, 1398-1407.

Steens, A., de, V.A., Hemmen, J., Heersema, T., Heerings, M., Maurits, N., Zijdewind, I., 2012. Fatigue perceived by multiple sclerosis patients is associated with muscle fatigue. Neurorehabil.Neural Repair 26, 48-57.

Stefancin, P., Govindarajan, S.T., Krupp, L., Charvet, L., Duong, T.Q., 2019. Resting-state functional connectivity networks associated with fatigue in multiple sclerosis with early age onset. Mult Scler Relat Disord 31, 101-105.

Tartaglia, M.C., Narayanan, S., Francis, S.J., Santos, A.C., De Stefano, N., Lapierre, Y., Arnold, D.L., 2004. The relationship between diffuse axonal damage and fatigue in multiple sclerosis. Arch Neurol 61, 201-207.

Tedeschi, G., Dinacci, D., Lavorgna, L., Prinster, A., Savettieri, G., Quattrone, A., Livrea, P., Messina, C., Reggio, A., Servillo, G., Bresciamorra, V., Orefice, G., Paciello, M., Brunetti, A., Paolillo, A., Coniglio, G., Bonavita, S., Di Costanzo, A., Bellacosa, A., Valentino, P., Quarantelli, M., Patti, F., Salemi, G., Cammarata, E., Simone, I., Salvatore, M., Bonavita, V., Alfano, B., 2007. Correlation between fatigue and brain atrophy and lesion load in multiple sclerosis patients independent of disability. J Neurol Sci 263, 15-19.

Tellez, N., Alonso, J., Rio, J., Tintore, M., Nos, C., Montalban, X., Rovira, A., 2008. The basal ganglia: a substrate for fatigue in multiple sclerosis. Neuroradiology 50, 17-23.

Tomasevic, L., Zito, G., Pasqualetti, P., Filippi, M., Landi, D., Ghazaryan, A., Lupoi, D., Porcaro, C., Bagnato, F., Rossini, P., Tecchio, F., 2013. Cortico-muscular coherence as an index of fatigue in multiple sclerosis. Mult Scler 19, 334-343.

van der Werf, S.P., Jongen, P.J., Lycklama a Nijeholt, G.J., Barkhof, F., Hommes, O.R., Bleijenberg, G., 1998. Fatigue in multiple sclerosis: interrelations between fatigue complaints, cerebral MRI abnormalities and neurological disability. J Neurol Sci 160, 164-170.

Vecchio, F., Miraglia, F., Porcaro, C., Cottone, C., Cancelli, A., Rossini, P.M., Tecchio, F., 2017. Electroencephalography-Derived Sensory and Motor Network Topology in Multiple Sclerosis Fatigue. Neurorehabilitation and Neural Repair 31, 56-64.

Wilting, J., Rolfsnes, H.O., Zimmermann, H., Behrens, M., Fleischer, V., Zipp, F., Groger, A., 2016. Structural correlates for fatigue in early relapsing remitting multiple sclerosis. Eur Radiol 26, 515-523.

Wolkorte, R., Heersema, D.J., Zijdewind, I., 2015a. Muscle Fatigability During a Sustained Index Finger Abduction and Depression Scores Are Associated With Perceived Fatigue in Patients With Relapsing-Remitting Multiple Sclerosis. Neurorehabil Neural Repair 29, 796-802.

Wolkorte, R., Heersema, D.J., Zijdewind, I., 2015b. Reduced Dual-Task Performance in MS Patients Is Further Decreased by Muscle Fatigue. Neurorehabil Neural Repair 29, 424-435.

Wolkorte, R., Heersema, D.J., Zijdewind, I., 2016. Reduced Voluntary Activation During Brief and Sustained Contractions of a Hand Muscle in Secondary-Progressive Multiple Sclerosis Patients. Neurorehabil Neural Repair 30, 307-316.

Yaldizli, O., Glassl, S., Sturm, D., Papadopoulou, A., Gass, A., Tettenborn, B., Putzki, N., 2011. Fatigue and progression of corpus callosum atrophy in multiple sclerosis. J Neurol 258, 2199-2205.

Yarraguntla, K., Bao, F., Lichtman-Mikol, S., Razmjou, S., Santiago-Martinez, C., Seraji-Bozorgzad, N., Sriwastava, S., Bernitsas, E., 2019. Characterizing Fatigue-Related White Matter Changes in MS: A Proton Magnetic Resonance Spectroscopy Study. Brain Sci 9.

Zaini, W.H., Giuliani, F., Beaulieu, C., Kalra, S., Hanstock, C., 2016. Fatigue in Multiple Sclerosis: Assessing Pontine Involvement Using Proton MR Spectroscopic Imaging. PLoS One 11.

Zellini, F., Niepel, G., Tench, C.R., Constantinescu, C.S., 2009. Hypothalamic involvement assessed by T1 relaxation time in patients with relapsing-remitting multiple sclerosis. Mult Scler 15, 1442-1449.
